# Combinatorial effects of *Zingiber officinale* and *Citrus limon* juices: Hypolipidemic and antioxidant insights from *in vivo*, *in vitro*, and *in silico* investigations

**DOI:** 10.1371/journal.pone.0319721

**Published:** 2025-09-12

**Authors:** Oussama Bekkouch, Ayoub Bekkouch, Hamza Elbouny, Ilham Touiss, Soufiane El Assri, Mohammed Choukri, Mohammed Bourhia, Kiryang Kim, Sojin Kang, Min Choi, Jinwon Choi, Hyo Jeong Kim, Chi-Hoon Ahn, Moon Nyeo Park, Bonglee Kim, Souliman Amrani

**Affiliations:** 1 Higher Institute of Nursing Professions and Health Techniques, Oujda, Morocco; 2 Laboratory of Bioresources, Biotechnology, Ethnopharmacology and Health, Faculty of Sciences, Mohammed First University, Oujda, Morocco; 3 Laboratory of Biology and Health, Faculty of Sciences, University Ibn Tofail, Kenitra, Morocco; 4 Laboratory of Biochemistry, Faculty of Sciences and Technology, University Moulay Ismail, Errachidia, Morocco; 5 Laboratory of Life and Health Sciences, Department of Pharmaceutical Sciences, Faculty of Medicine and Pharmacy, University Abdelmalek Essaadi, Tangier, Morocco; 6 Laboratory of Biochemistry, University Hospital Center Mohammed VI, Oujda, Morocco; 7 Faculty of Medicine and Pharmacy, Mohammed First University, Oujda, Morocco; 8 Laboratory of Biotechnology and Natural Resources Valorization, Faculty of Sciences, Ibn Zohr University, Agadir, Morocco; 9 Department of Pathology, College of Korean Medicine, Kyung Hee University, Seoul, Republic of Korea; Faculty of Pharmaceutical sciences Times institute Multan, PAKISTAN

## Abstract

The combined use of *Zingiber officinale* (ginger) and *Citrus limon* (lemon) has been traditionally recognized for its therapeutic potential, particularly in promoting cardiovascular health. However, the synergistic effects of these botanicals on hyperlipidemia and oxidative stress, two major contributors to cardiovascular disease, remain insufficiently characterized. This study employed an integrative approach involving *in vivo*, *in vitro*, and *in silico* methodologies to assess the hypolipidemic and antioxidant properties of ginger and lemon juices. *In vivo* experiments were conducted using a murine model of Triton WR-1339-induced hyperlipidemia. Antioxidant activity was evaluated through DPPH and ABTS assays, while molecular docking simulations were performed to assess interactions with HMG-CoA reductase, a key enzyme in cholesterol metabolism. Phytochemical profiling revealed high levels of bioactive compounds, with ginger juice containing 6-gingerol (15.54% peak area), and lemon juice rich in hesperidin (13.85%) and rutin (5.57%). These constituents likely contributed to the observed pharmacological effects. The combined formulation led to marked improvements in lipid parameters, including reductions in total cholesterol (−54.3%), triglycerides (−49.8%), and LDL-C (−58.1%), alongside an increase in HDL-C (+47.6%) compared to hyperlipidemic controls (p < 0.05). Antioxidant assays further demonstrated strong free radical scavenging activity, with IC₅₀ values of 30.92 ± 2.00 µg/mL (ABTS) and 44.94 ± 1.02 µg/mL (DPPH) for the combined formulation. Molecular docking confirmed high binding affinities of key compounds, with hesperidin and 6-gingerol displaying binding energies of −10.4 and −9.6 kcal/mol, respectively, against HMG-CoA reductase. In summary, the combination of *Zingiber officinale* and *Citrus limon* juices exhibited potent lipid-lowering and antioxidant activities, outperforming the effects observed with individual extracts. These findings underscore their potential as natural therapeutic agents for the management of hyperlipidemia and oxidative stress. Further clinical investigations are warranted to validate these preclinical results and to explore their future application in the development of nutraceuticals or functional foods.

## 1 Introduction

Lipids play a crucial role in regulating numerous cellular physiological functions, and changes in membrane lipid metabolism are linked to significant diseases such as cancer, type II diabetes, cardiovascular disease, and immune disorders [1]; however, an elevated presence of plasmatic fats heightens the susceptibility to coronary heart disease. Dyslipidemia is a spectrum of abnormalities in lipoproteins and lipids, a primary risk factor for atherosclerosis [2]. Thus, it becomes imperative to lower lipid levels in individuals with hyperlipidemia.

Several lipid-lowering drugs in hyperlipidemia therapy include statins and fibrates [3]. Regrettably, synthetic drugs designed to combat hyperlipidemia and atherosclerosis may entail multiple adverse effects [3,4]. Phytotherapy is a safe solution; herbal medicine may be a suitable alternative to conventional medicine for treating several disorders, including hyperlipidemia and cardiovascular disease [5]. Additionally, more solutions and products must be found and developed in addition to conventional medications to control lipid levels and prevent cardiovascular disease [6].

In recent years, scientific interest has increasingly turned toward optimizing the therapeutic potential of plant-derived compounds through advanced formulation strategies. Ginger (*Zingiber officinale*), long celebrated for its antioxidant and lipid-lowering properties, has now been incorporated into modern delivery systems such as silver nanoparticles and niosomal gels, an innovation that bridges traditional herbal medicine with contemporary nanotechnology. These novel formulations exhibit superior efficacy compared to crude extracts, mainly due to enhanced surface-area-to-volume ratios, improved permeability, and more sustained bioavailability. Ongtanasup et al., (2024a) demonstrated that green-synthesized silver nanoparticles derived from *Zingiber officinale* possess markedly improved antioxidant and anti-lipoxygenase (LOX) activities, suggesting a more targeted modulation of oxidative stress pathways [7]. Complementarily, the use of niosomal red palm wax gels encapsulating ginger has proven effective in both preclinical and early clinical settings, supporting its therapeutic viability in metabolic conditions [8]. Integrating such approaches into the development of functional botanical formulations reflects an exciting and much-needed evolution in the field of phytotherapy.

Ginger, scientifically known as *Zingiber officinale* Roscoe, was identified by English botanist William Roscoe and belongs to the Zingiberaceae family. Native to South Asia, this plant possesses perennial rhizomes and thrives in subtropical and tropical regions, scaling altitudes up to 1500 meters above sea level [9]. *Zingiber officinale* has been a globally utilized spice and herb, notably in India and China, the primary ginger producers. It assumes a crucial role in traditional Chinese herbal medicine and is believed to have spread to other continents during the Roman and Greek epochs [10]. *Zingiber officinale* has also been extensively applied to treating diverse diseases, including indigestion, nausea, asthma, and muscle pain [11].

*Citrus limon* L., or lemon, is a plant species belonging to the Rutaceae family that grows in sub-tropical and Mediterranean climates. The *Citrus limon* tree produces a fruit (lemon) widely used for its juice, mainly as a condiment [12]. The juice of this fruit has an acidic flavor and a low pH (2–3) due to its richness in organic acids [13]. Numerous pharmacological effects of *Citrus limon* have been demonstrated, including antioxidant [14], anti-inflammatory [15], hepatoprotective [16], diabetes prevention and anti-obesity [17], and lipolytic and cholesterol-lowering [18] effects.

This study investigates the anti-hyperlipidemic impact of *Zingiber officinale* and *Citrus limon* juices, individually and in combination, on triton-induced hyperlipidemic mice.

Indeed, while significant strides have been made in understanding the therapeutic potential of natural compounds in the context of hyperlipidemia, a conspicuous gap remains in the literature concerning the combined effect of *Zingiber officinale* and *Citrus limon* juice extracts on Triton WR-1339-induced acute hyperlipidemia. Our study aimed to bridge this gap by investigating the synergistic impact of these two botanical extracts in a formulated treatment. Furthermore, a comprehensive phytochemical analysis was conducted to identify and characterize the bioactive compounds present in the extracts. Recognizing the pivotal role of HMG-CoA reductase in cholesterol metabolism, a molecular docking study was undertaken to elucidate the potential interactions between the bioactive compounds and the enzyme. Our research sheds light on the therapeutic efficacy of the formulated juice extract and contributes valuable insights into the molecular mechanisms underlying its effects.

To our knowledge, this is the first study to evaluate the combined hypolipidemic effect of *Zingiber officinale* and *Citrus limon* juices in an *in vivo* hyperlipidemic model, complemented by antioxidant assays and a molecular docking analysis. This approach and focus distinguish our work from previous studies on these plants (e.g., their hepatoprotective effects in a chronic liver injury model), thereby providing new insights into their synergistic lipid-lowering potential.

## 2 Materials and methods

### 2.1. Chemicals

The following reagents were purchased from Sigma Chemical Co. (Taufkirchen, Germany): Triton WR-1339, Folin-Ciocalteu, Sodium Carbonate (Na_2_CO_3_), Gallic Acid, Sodium Nitrate (NaNO_3_), Aluminum Chloride (AlCl_3_), Sodium Hydroxide (NaOH), Quercetin, Sodium Phosphate (Na_3_PO_4_).

### 2.2. *Zingiber officinale* Roscoe (Ginger)

*Zingiber officinale* Roscoe (Ginger) rhizomes at the mature stage were purchased from an herbalist (Oujda, Morocco) and well-cleaned with distilled water to remove any dirt. Professor Fennane Mohammed, a qualified botanist from the Scientific Institute of Rabat (Morocco), identified the plant material. A reference voucher specimen with the reference number was kept in the herbarium of Mohamed First University’s Faculty of Sciences in Oujda, Morocco (HUMPOM-352).

### 2.3. *Citrus limon* L. (Lemon)

At the mature stage, the lemon fruits (*Citrus limon* L.) were purchased from a local market (Oujda, Morocco), washed adequately with distilled water, and authenticated by Professor Fennane Mohammed. A reference voucher specimen has been deposited in the herbarium of the Faculty of Sciences of Mohamed First University (Oujda, Morocco) under the reference number (HUMPOM-450).

### 2.4. *Zingiber officinale* and *Citrus limon* juice extraction

#### 2.4.1. *Zingiber officinale* juice extraction.

Since *Zingiber officinale* rhizomes are naturally rich in water, no external solvent was added; the intracellular water served as the sole extraction medium.

To make ginger juice (GJ), 500 g of fresh ginger rhizomes were cut into smaller pieces (approximately 1 cm) and ground in a household blender at 25 °C. Since ginger rhizomes are naturally rich in water, no external solvent was added; the intracellular water served as the sole extraction medium. The homogenized mixture was then filtered through Whatman Grade 40 high-purity filters (nominal particle retention of 8 µm) to remove fibrous residues, yielding a clear, aqueous ginger extract. The same protocol was applied to lemon juice (LJ) preparation using fresh lemons, with no solvents added and concentrated using a rotating vacuum evaporator (Heidolph Rotary Evaporator, Laborota 4010 intensive condenser G4) before being stored at −20 °C until its use.

#### 2.4.2. *Citrus limon* juice extraction.

Lemons were washed and cut into small pieces weighing 500 g before being ground for 1–2 minutes using a blender to extract the lemon juice (LJ). Then, the same procedures were performed precisely as those for ginger juice extraction, using no added solvents.

#### 2.4.3. Formulation preparation.

The formulation was prepared by combining 50% *Zingiber officinale* juice (GJ) with 50% *Citrus limon* juice (LJ), a 1:1 ratio deliberately chosen to reflect everyday daily use, where these juices are typically mixed in equal parts for their complementary taste and reputed health benefits. In this study, we employed a solvent-free juice extraction method to preserve the native bioactivity of the compounds. By avoiding high temperatures and chemical solvents, this approach promotes safer, more sustainable, and environmentally responsible preparation while maintaining the natural integrity of the bioactive constituents. The resulting mixture was lyophilized to obtain a homogeneous powder, which was then used to prepare the desired concentrations for experimental application.

### 2.5. Phytochemical analysis

The phytochemical properties of *Zingiber officinale* and *Citrus limon* were analyzed to identify lead compounds and components associated with the observed physiological effects. The distinctive biological activities of these plants can be determined through their phytochemical properties.

The phytochemical studies done in this work were: “The total phenolic quantification”, “the total flavonoids determination”, and “the HPLC analysis”.

#### 2.5.1. Total phenolics quantification.

The quantification of total phenolics in *Zingiber officinale* and *Citrus limon* juices was carried out using a slightly modified method as described by [19], and the calculation of the amount of the polyphenol was based on a calibration curve of the gallic acid. Dilutions of each juice (500 µL) were combined with 2,500 µL of Folin-Ciocalteu reagent (0.2 N) and 2,000 µL of sodium carbonate (Na_2_CO_3_) solution (7.5% w/v) at 25 °C. After 15 minutes of incubation in the dark, absorbance was measured at 765 nm. The process for the blank was the same, except that solvent was used instead of juice. Total phenolic content was expressed in micrograms of gallic acid equivalents per milligram of extract (mg GAE/mg extract) using a calibration curve prepared with gallic acid.

#### 2.5.2. Total flavonoids determination.

The determination of flavonoids in *Zingiber officinale* and *Citrus limon* juices were conducted following the method outlined by [20], and the calculation of the flavonoid amount was based on a calibration curve of quercetin. Dilutions of each juice (200 µL) were mixed with 1 mL of distilled water and 50 µL of sodium nitrate (NaNO_3_) solution (5% w/v). The mixture was then combined and left to incubate at room temperature for 5 minutes. Subsequently, 120 µL of aluminum chloride (AlCl_3_) solution (10% w/v) was added. After another 5 minutes of incubation at 25 °C, 400 µL of sodium hydroxide (NaOH) solution (1M) was introduced to the reaction, followed by absorbance measurement at 430 nm. The procedure for preparing the blank was identical, except that solvent was used instead of juice. The total flavonoid content was quantified in micrograms of quercetin equivalents per milligram extract (mg E.Q./mg).

#### 2.5.3. High-Performance Liquid Chromatography (HPLC).

The ginger and lemon juice extracts were analyzed using high-performance liquid chromatography (HPLC) to identify major constituents. Chromatographic separation was performed on a reversed-phase C18 column (250 × 4.6 mm, 5 µm) with a suitable gradient mobile phase (solvent A: water + 0.1% formic acid; solvent B: acetonitrile + 0.1% formic acid).

The HPLC system (Waters Alliance 2695, Milford, MA, USA) was used in addition to a range of standards, including 4-gingerol, 6-gingerol, 6-gingediol, eriodictyol, rutin, hesperidin, and isorhamnetin.

The eluent flow rate was kept at 1 mL/min, and the injection volume was set at 20 μL. All analyses were conducted at room temperature.

Standard and extract solutions of *Zingiber officinale* and *Citrus limon* (GJ, LJ, and F) were dissolved in methanol and filtered through a 0.45 μm Millipore membrane.

### 2.6. Antioxidant study of *Zingiber officinale* and *Citrus limon* Juices

#### 2.6.1. Phosphomolybdenum assay.

The total antioxidant capacity (TAC) was evaluated using the phosphomolybdenum method, following the previously described method [21] was meticulously adopted to assess the total antioxidant capacity (TAC) of the extract, employing a well-established protocol. Specifically, 0.1 mL of the extract was combined with a reagent mixture containing 0.6 M sulfuric acid, 28 mM sodium phosphate, and 4 mM ammonium molybdate in a reaction tube. The tubes were securely sealed and incubated in a water bath at 95°C for 90 minutes to facilitate the reaction. Following incubation, the absorbance of the solutions was measured at 695 nm against a reagent blank. The results were quantified as milligrams of ascorbic acid equivalents per gram of dry weight (mg AAE g ⁻ ¹ DW), providing a standardized measure of the extract’s antioxidant capacity.

#### 2.6.2. ABTS assay.

The radical-scavenging activity of ABTS [2,2′-azino-bis(3-ethylbenzothiazoline-6-sulfonic acid)] was determined using the method established by Re et al. (1999) [22], with slight modifications. Briefly, an aqueous solution of potassium persulfate (2.45 mM) was mixed with a 7 mM ABTS aqueous solution to prepare the assay reagent, which was then left in the dark at room temperature overnight to allow radical generation. Before analysis, the reagent was diluted with ethanol (EtOH) to obtain an absorbance of 0.70 (± 0.01) at 734 nm. For the assay, 100 µL of the extract at varying concentrations was added to 2 mL of the ABTS solution. The mixture was incubated for 10 minutes, and absorbance was measured at 734 nm.

Ethanol served as the control, while calibration was performed using a standard curve of ascorbic acid solutions. The percentage inhibition was calculated using the formula:


$$𝐀𝐁𝐓𝐒\;𝐫𝐚𝐝𝐢𝐜𝐚𝐥\;𝐢𝐧𝐡𝐢𝐛𝐢𝐭𝐢𝐧𝐠\;𝐚𝐜𝐭𝐢𝐯𝐢𝐭𝐲\;\left(\%\right)=\left(\frac{\text{Ac}-\text{At}}{\text{Ac}}\right)\text{x 100}$$

Where “Ac” represents the absorbance of the control (without the extract) and “At” represents the absorbance of the test sample.

Results were expressed as IC_50_ values, indicating the sample concentration required to inhibit 50% of the ABTS radicals.

#### 2.6.3. DPPH radical scavenging assay.

Thanks to its stability as a free radical and the simplicity of its analysis, DPPH (2,2-diphenyl-1-picrylhydrazyl) has become a popular tool for quickly and efficiently evaluating antioxidant activity [23]. Subhashini et al. (2011) detailed the experimental procedure to explore DPPH’s radical scavenging ability [24]. Specifically, 1 mL of the plant’s etheric extract was mixed with 1 mL of a DPPH radical solution, achieving a final DPPH concentration of 0.025 g/L. After thorough stirring, the mixture was left to rest for 30 minutes, and its absorbance was then recorded at 517 nm. Ascorbic acid served as the standard reference. The percentage of DPPH radical inhibition was calculated using an equation, as outlined by Manzocco et al. (1998) [25].


$$𝐏𝐞𝐫𝐜𝐞𝐧𝐭𝐚𝐠𝐞\;𝐨𝐟\;𝐢𝐧𝐡𝐢𝐛𝐢𝐭𝐢𝐨𝐧\;\left(\%\right)=\left(\frac{\text{Abs Control}-\text{Abs Sample}}{\text{Abs Control}}\right)\text{x 100}$$

### 2.7. Evaluation of the protective effect of GJ, LJ, and BHA against plasma lipoprotein oxidation

The protective effects of ginger juice (GJ), lemon juice (LJ), and butylated hydroxyanisole (BHA) against plasma lipoprotein oxidation were assessed by quantifying malondialdehydes (MDA), secondary products of lipoprotein oxidation, as thiobarbituric acid reactive substances (TBARS), following the method described by Bekkouch et al. (2019) [26].

Lipoprotein-rich plasma, used as the oxidation substrate, was collected from mice treated with 400 mg/kg of Triton WR-1339 for 10 hours. As previously determined, this plasma contained approximately 100 ± 5 mg/dL of LDL cholesterol.

The oxidative process was initiated using copper sulfate (CuSO₄) according to the following protocol:

- **Control group**: 40 µL of lipoprotein-rich plasma incubated with distilled water.
- **Oxidized lipoproteins group**: 40 µL of lipoprotein-rich plasma incubated with 10 µL of CuSO₄ (0.3 mg/mL).
- **GJ-treated lipoproteins group**: 40 µL of lipoprotein-rich plasma incubated with 10 µL of CuSO₄ solution and *Zingiber officinale* juice at concentrations of 5, 12.5, 25, 50, 100, and 200 µg/mL.
- **LJ-treated lipoproteins group**: 40 µL of lipoprotein-rich plasma incubated with 10 µL of CuSO₄ solution and *Citrus limon* juice at concentrations of 5, 12.5, 25, 50, 100, and 200 µg/mL.
- **Formulation-treated lipoproteins group**: 40 µL of lipoprotein-rich plasma incubated with 10 µL of CuSO₄ solution and ginger and lemon Formulation at concentrations of 5, 12.5, 25, 50, 100, and 200 µg/mL.
- **BHA-treated lipoproteins**: 40 µL of lipoprotein-rich plasma incubated with 10 µL of CuSO₄ solution and BHA at concentrations of 5, 12.5, 25, 50, 100, and 200 µg/mL.

After vigorous mixing, the preparations were incubated at 37°C for 24 hours. Subsequently, 500 µL of 20% trichloroacetic acid and 500 µL of 0.8% thiobarbituric acid were added to each sample. The reaction mixtures were heated at 95°C for 30 minutes, then cooled to room temperature. The absorbance was measured at 532 nm.

TBARS levels were calculated and expressed as MDA equivalents, determined from a calibration curve. All measurements were performed in triplicate to ensure accuracy and reliability.

### 2.8. Hypolipidemic effect of *Zingiber officinale* and *Citrus limon* juices in mice model

#### 2.8.1. Animals.

90 adult male albino mice weighing 25–30 g were raised in the biology department’s animal house at the Faculty of Sciences in Oujda, following international guidelines established by the National Institutes of Health of the United States (NIH Publication No. 85–23, revised 1985) for the protection and usage of laboratory animals [27]. The Faculty of Sciences Institutional Review Board of Oujda University, Morocco, approved the study (03/21-LBBEH-15 and March 5, 2021).

The animals were maintained in a temperature-controlled room (22 ± 2°C) with a 12-hour light/12-hour dark cycle and provided food and water *ad libitum*.

We purposely used only male mice to minimize variability due to hormonal cycles and sex-specific lipid metabolism, as male rodents consistently exhibit higher and more stable plasma lipid levels compared to females.

#### 2.8.2. Induction of hyperlipidemia by triton WR-1339.

Triton WR-1339 is a nonionic detergent widely used to induce acute hyperlipidemia in animal models to study cholesterol and triglyceride metabolism [6]. The accumulation of plasma lipids caused by this detergent is due to the inhibition of lipoprotein lipase activity [28]. Animals were injected with a dose of 200 mg/kg, according to [29].

Adult male *Albino* mice were divided into nine groups of 10 animals each:

- **Group 1:** normolipidemic control animals gavaged with distilled water (NCG).
- **Group 2:** animals receiving Triton WR-1339 (200 mg/kg in 9‰ NaCl; pH = 7.4) by intraperitoneal injection and gavaged with distilled water (HCG).
- **Group 3:** Animals injected with Triton WR-1339 (200 mg/kg) and administered with *Z. officinale* juice (250 mg/kg) (GJTG 1).
- **Group 4:** Animals injected with Triton WR-1339 (200 mg/kg) and treated with *Z. officinale* juice (500 mg/kg) (GJTG 2).
- **Group 5:** Animals injected with Triton WR-1339 (200 mg/kg) and treated with *C. limon* juice extract (250 mg/kg) (LJTG 1).
- **Group 6:** Animals are given Triton WR-1339 (200 mg/kg) and administered with *C. limon* juice extract (500 mg/kg) (LJTG 2).
- **Group 7:** Animals receiving Triton WR-1339 (200 mg/kg) and treated with the formulation of the *Z. officinale* and the *C. limon* juices (250 mg/kg) (FTG 1).
- **Group 8:** Animals fed Triton WR-1339 (200 mg/kg) and gavaged with the formulation from *Z. officinale* juice and *C. limon* juices (500 mg/kg) (FTG 2).
- **Group 9:** animals receiving Triton WR-1339 (200 mg/kg) and gavaged with Atorvastatin (10 mg/kg) (ATG).

#### 2.8.3. Biochemical analysis.

a) Blood Sampling

Twenty-four hours after injection, mice were anesthetized using a combination of ketamine (80 mg/kg) and xylazine (10 mg/kg) to ensure adequate sedation and minimize discomfort prior to blood collection. Blood was obtained via the retro-orbital plexus using the retro-orbital sampling technique, which is particularly suitable for efficient and repeated blood drawings in studies requiring multiple time points. The collected blood samples were placed into heparinized Eppendorf tubes and centrifuged at 1500 rpm for 15 minutes to separate the plasma. The resulting plasma fractions were carefully transferred into new tubes and stored for subsequent lipid analysis.

b) Animal experiments

All experimental procedures were performed in strict accordance with ethical standards aimed at minimizing animal distress. Measures included the use of ketamine–xylazine anesthesia during invasive interventions, gentle and atraumatic handling, and continuous monitoring of animal welfare throughout the study period.

Additionally, an official ethics approval certificate was granted by our institutional ethics committee, confirming that all protocols were thoroughly reviewed and approved in line with internationally recognized guidelines. Comprehensive efforts were made at every stage of the study to ensure the well-being of the animals and to minimize potential discomfort and suffering.

c) Determination of Total Cholesterol (TC)

Plasma total cholesterol (TC) levels were determined using an enzymatic colorimetric method with a commercial assay kit (Biosystems, Barcelona, Spain; REF: 12505). Briefly, 1 mL of the enzymatic reagent was added to 10 µL of the plasma sample and incubated for 10 minutes at 37 °C. The absorbance was then measured at 510 nm using a spectrophotometer. Cholesterol concentrations were calculated according to the manufacturer’s instructions, and all measurements were performed in duplicate to ensure accuracy and reproducibility.

d) Determination of Triglycerides (TG)

Plasma triglycerides were determined using a commercial kit (Biosystems Kit, Barcelona, Spain, REF: 12558). The enzymatic triglyceride reagent was mixed with 10 µL of plasma samples for 10 min at 37°C. The absorbance was then determined at 510 nm, and the TG was determined according to the manufacturer’s instructions.

e) High-Density Lipoprotein Cholesterol (HDLc) and Low-Density Lipoprotein Cholesterol (LDLc) assay

High-density lipoprotein cholesterol (HDL-C) was quantified following the selective precipitation of low-density (LDL) and very-low-density lipoproteins (VLDL) using a phosphotungstic acid (PTA) and magnesium chloride (MgCl₂) reagent. Briefly, 20 µL of plasma was mixed with 10 µL of PTA/MgCl₂ solution and allowed to stand for 10 minutes before centrifugation at 5,000 rpm for 15 minutes. The resulting supernatant, containing the HDL fraction, was then used for enzymatic cholesterol determination, following the same procedure as for total cholesterol analysis.

Low-density lipoprotein cholesterol (LDL-C) concentrations were calculated using the Friedewald formula.

### 2.9. Acute toxicity study in mice

The acute oral toxicity study was conducted per the Organization for Economic Cooperation and Development (OECD) guidelines 423 [30].

96 mice were used, divided into 16 groups of 6 mice each (3 males and three females per group). The first group served as the control and received only distilled water. The remaining 15 groups were administered increasing doses of the GJ, LJ, and formulation F at 2, 4, 6, 8, and 10 g/kg of body weight.

Following the oral administration of the GJ, LJ, and formulation F, the mice were carefully observed individually for the first 30 minutes. Regular monitoring continued over the next 24 hours, with special attention given to the first 4 critical hours. This observation period was maintained daily for 14 days to thoroughly assess potential harmful effects.

### 2.10. Subacute toxicity study

#### 2.10.1. Animal protocol.

Eleven groups of rats were created to assess subacute toxicity, each with six individuals (three males and three females). The groups were organized as follows:

a) Control Group: Received distilled water orally for 30 days.
b) Test Groups: Administered different doses of each extract (500, 1000, or 2000 mg/kg body weight) by gavage daily for 30 days.

The dose levels were carefully determined based on the LD_50_ and guidelines provided in the Organisation for Economic Cooperation and Development (OECD) document 407.

Throughout the 30 days, the animals were monitored daily to check for any changes in their general health or signs of toxicity. Body weight measurements were taken on days 0, 7, 14, 21, and 28 to track fluctuations.

At the end of the study, the animals fasted overnight before blood samples were collected under anesthesia from the abdominal aorta. Blood was drawn into two types of tubes:

- **EDTA-coated tubes:** These were used immediately for hematological analysis.
- ***Plain tubes*:** These were centrifuged at 3000 rpm at 4°C for 10 minutes to separate the serum, which was later used for biochemical testing.

#### 2.10.2. Serum biochemistry.

To gain a deeper understanding of the physiological impacts of the treatment, we analyzed serum samples using an automated chemistry analyzer (COBAS INTEGRA^®^ 400 Plus). This process allowed us to measure various important biochemical markers that reflect the health of key organs. Albumin (ALB) levels were assessed as a general indicator of liver function and nutritional status. Alkaline phosphatase (ALP) was analyzed to check for potential liver or bone health issues. Furthermore, we measured alanine aminotransferase (ALT) and aspartate transaminase (AST), two enzymes commonly used to detect liver stress or damage.

We also evaluated bilirubin (BIL), which can signal liver function abnormalities or hemolytic activity. To assess lipid metabolism, cholesterol (CHOL) and triglycerides (TRGL) were measured, providing insights into the subjects’ cardiovascular and metabolic health. Finally, creatinine (CRE) and urea (URE) were analyzed to gauge kidney function and overall protein metabolism.

Combining all these parameters provided a detailed picture of how the treatment affected the animals’ internal systems. This comprehensive approach helped us identify potential toxic effects, particularly on the liver, kidneys, and metabolic functions, ensuring a thorough evaluation of the treatment’s safety.

#### 2.10.3. Hematologic markers.

Hematological analysis was performed using an automated analyzer (Abacus 380 Hematology Analyzer) to generate a detailed profile of blood composition. The assessment encompassed key parameters reflective of systemic and hematological health.

White blood cell (WBC) counts were measured to assess immune function, while red blood cell (RBC) counts provided information on the blood’s oxygen-carrying capacity. Hemoglobin (HGB) concentration and hematocrit (HCT) values were evaluated to further characterize oxygen transport and total blood volume.

To gain deeper insights into erythrocyte characteristics, red cell indices including mean corpuscular volume (MCV), mean corpuscular hemoglobin (MCH), and mean corpuscular hemoglobin concentration (MCHC) were analyzed. These indicators offered a refined view of red cell morphology and hemoglobin distribution.

Platelet (PLT) counts, and mean platelet volume (MPV) were also measured to evaluate thrombocyte abundance and platelet size, providing indirect markers of hemostatic function and platelet activity.

Together, this comprehensive hematological assessment allowed for a robust evaluation of the treatments’ potential effects on the cellular components of blood and ensured a thorough understanding of systemic hematological responses.

### 2.11. Molecular docking study

Following the demonstration of the cholesterol-lowering effects of *Zingiber officinale* and *Citrus limon* juices, an *in silico* investigation was undertaken to further explore the underlying molecular mechanisms, particularly the signaling pathways involved. This computational analysis aimed to evaluate the binding affinity of the most abundant bioactive compounds from both extracts with the key enzyme in cholesterol biosynthesis, the 3-hydroxy-3-methylglutaryl-coenzyme A reductase (HMG-CoA reductase).

Molecular docking simulations were performed to assess the interactions between the major identified phytochemicals and the enzyme. For this purpose, the X-ray crystal structure of the catalytic domain of human HMG-CoA reductase (PDB ID: 1DQ9) was retrieved and employed as the receptor model [31]. AutoDock Vina (ver. 1.1.2) [32] was employed for docking simulations [33]. Protein preparation involved removing the native ligand (HMG-CoA) from the enzyme’s binding pocket, followed by the addition of polar hydrogen and assignment of Gasteiger charges to the receptor. The binding site was defined based on the active site cavity, with a grid box of 40 × 40 × 40 Å³ centered on the HMG-CoA binding region to provide sufficient space for ligand interaction.

Ligand structures, including 6-gingerol, 4-gingerol, 6-gingediol, hesperidin, rutin, eriodictyol, isorhamnetin, and Atorvastatin (used as a reference), were subjected to energy minimization and converted into the appropriate input format. Molecular docking was carried out using an exhaustive value of 8, generating ten binding poses for each compound. The pose with the most favorable binding affinity (i.e., the lowest ΔG value in kcal/mol) was selected for analysis. Binding energies calculated via AutoDock Vina were used to evaluate and compare the predicted inhibitory potential of each compound. [32]. The binding between the enzyme and the molecules occurred thanks to the AutoDock software, which allowed us to determine the affinity between the enzyme and each molecule. Then, the critical points were visualized in 2D thanks to the Biovia Discovery Studio software.

### 2.12. Statistical analysis

The *Student*’s t-test was used to analyze the collected experimental data. GraphPad Prism 9.0.0 (GraphPad Prism Software, Inc., San Diego, CA, USA) was used to perform an unpaired Student’s t-test for statistical significance between the two groups. Then, the analysis of variance (ANOVA) was followed by Tukey’s pairwise comparison test at a 95% confidence interval (p < 0.05). The P values were considered statistically significant because they were lower than 0.05. The results are shown as mean ± SEM.

## 3. Results

### 3.1. Extraction

The extraction yields were calculated based on the initial fresh weight of the plant materials. The obtained yields were 2.3% (w/w) for *Zingiber officinale* juice and 2.0% (w/w) for *Citrus limon* juice.

### 3.2. Determination of total phenolic content

**Fig 1:**
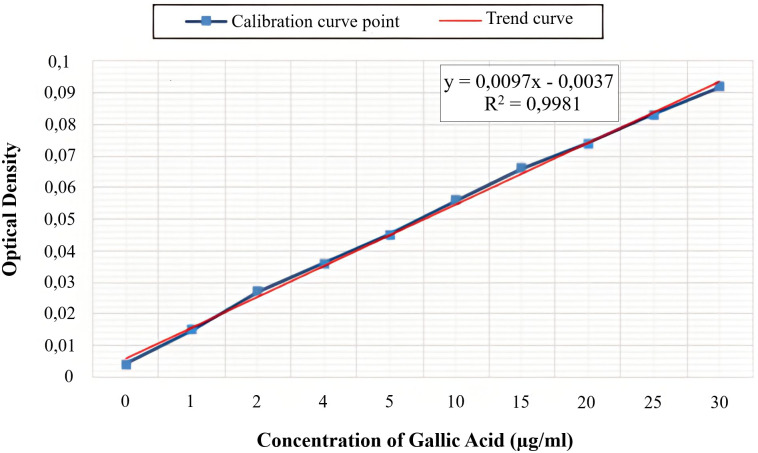
Standard calibration curve of Gallic Acid (GA) used for the quantification of total phenolic content. Data are presented as absorbance versus concentration (μg/mL). Linear regression and R² values are shown to assess the curve’s fit.

The determination of the total phenolic content by the method of Folin-Ciocalteu revealed the presence of 18.48 ± 1.14 mg and 25.23 ± 1.54 mg equivalent of gallic acid equivalent/g DW in the juices of *Z. officinale* juice (GJ) and *C. limon* juice (LJ) respectively (Table 1), the calculation of these quantities was due to a calibration curve of the gallic acid (Fig 1).

**Table 1:** Total phenolics and flavonoids quantification of ginger juice (GJ) and lemon juice (LJ).

	Total Phenolics (mg GAE/g DW)	Total Flavonoids (mg QE/g of DW)
**GJ**	18.48 ± 1.14	7.26 ± 2.05 ^b^
**LJ**	25.23 ± 1.54 ^a^	12.752.10 ^a^

### 3.3. Determination of total flavonoids content

The total flavonoid content revealed that “GJ” and “LJ” contain 7.26 ± 2.05 and 12.75 ± 2.10 mg equivalent of quercetin/g, respectively (Table 1). These quantities were calculated due to a calibration curve of the gallic acid (Fig 1).

**Fig 2:**
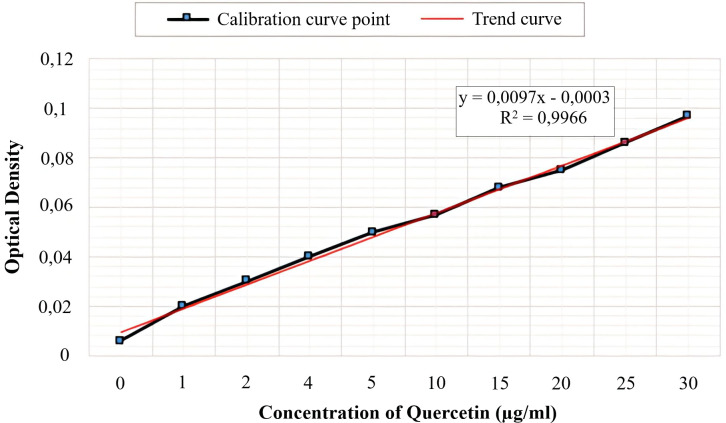
Standard calibration curve of Quercetin used for the quantification of flavonoids content. Data are presented as absorbance versus concentration (μg/mL). Linear regression and R² values are shown to assess the curve’s fit.

Columns with similar alphabets (letters a or b) are not significantly different (p < 0.05). Values are expressed as means ± SEM from triplicates. mg GAE/g DW: milligram equivalent of gallic acid equivalent per gram of dry weight, mg QE/g of DW: milligram equivalent of quercetin equivalent per dry weight (Fig 2).

### 3.4. HPLC chromatography of *Zingiber officinale* and *Citrus limon* juices

The results of the analysis of *Zingiber officinale* (GJ) and *Citrus limon* (LJ) juices using High-Performance Liquid Chromatography (HPLC) revealed distinct profiles for each.

**Fig 3:**
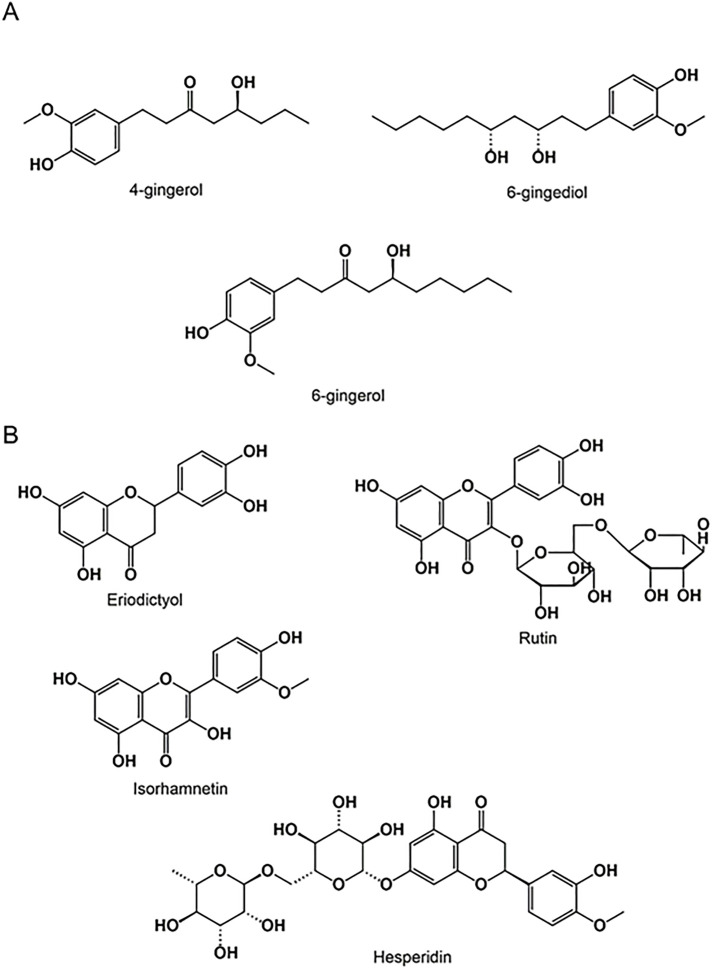
Chemical structure of *Zingiber officinale* (A) and *Citrus limon* (B) phenolic compounds (drawn using “ChemDraw 12.0”).

**Fig 4:**
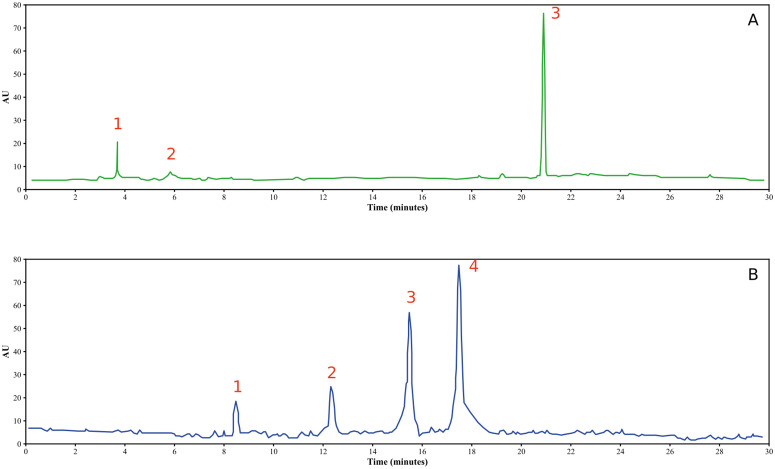
HPLC chromatographic profiles of *Zingiber officinale* (A) and *Citrus limon* (B) juices.

In the case of “GJ,” the main compounds identified were 6-gingerol, 4-gingerol, and 6-gingediol (Fig 3-A, Fig 4-A, Table 2).

**Table 2:** Chemical composition of *Zingiber officinale* juice (a) and **Citrus* *limon** (b).

Peak number	Compound	Retention time (min)	% of area
**(a)**			
**1**	4-gingerol	3.72	0.86
**2**	6-gingediol	6.55	0.22
**3**	6-gingerol	21.09	15.54
**(b)**			
**1**	Eriodictyol	9.33	3.21
**2**	Rutin	13.18	5.57
**3**	Hesperidin	16.70	13.85
**4**	Isorhamnetin	18.98	18.69

The HPLC analysis of “LJ” showed the existence of several molecules, and this analysis allowed us to prove the existence of hesperidin, rutin, isorhamnetin, and eriodictyol (Fig 3-B, Fig 4-B, Table 2).

On the other hand, the HPLC analysis of *C. limon* juice exhibited a more diverse range of compounds. This thorough analysis confirmed the presence of hesperidin, rutin, isorhamnetin, and eriodictyol in the *Citrus limon* juice (Fig 3-B, Fig 4-B, Table 2).

### 3.4. Antioxidant study of *Zingiber officinale* and *Citrus limon* juices

Table 3 elucidates the results of the antioxidant activities of *Zingiber officinale* (ginger) *and Citrus limon* (lemon) extracts across four antioxidant assays: DPPH, ABTS, TAC, and FRAP. The data demonstrates the effectiveness of the extracts, and their formulation (F) compared to ascorbic acid, a standard antioxidant. Lower IC_50_ values correspond to higher antioxidant activity.

**Table 3:** IC_50_ values for antioxidant activity of ginger, lemon, and their formulation across DPPH, ABTS, TAC, and FRAP assays. Lower IC_50_ values indicate stronger antioxidant potential. Ascorbic acid served as a reference compound.

	Ginger juice (GJ)	Lemon juice (LJ)	Formulation (F)	Ascorbic acid
**DPPH**	38.91 ± 1.47	43.14 ± 1.58	44.94 ± 1.02	40.11 ± 0.76
**ABTS**	31.78 ± 0.63	33.1 ± 1.74	30.92 ± 2.0	34.27 ± 1.39
**TAC**	58.43 ± 2.2	59.49 ± 2.34	63.9 ± 1.11	60.14 ± 0.77
**FRAP**	74.59 ± 1.85	76.91 ± 2.14	77.9 ± 1.76	75.12 ± 2.24

Data are expressed in (Mean ± SEM)

The DPPH free radical scavenging assay revealed that ascorbic acid exhibited the strongest antioxidant activity, with an IC₅₀ value of 40.11 ± 0.76 µg/mL. The combined juice formulation followed closely, showing an IC₅₀ of 44.94 ± 1.02 µg/mL. Interestingly, lemon juice had a slightly higher IC₅₀ (43.14 ± 1.58 µg/mL) compared to ginger juice (38.91 ± 1.47 µg/mL), suggesting that both juices possess similar radical-scavenging capacities. The improved performance of the combined formulation may reflect a synergistic interaction among its bioactive components (Table 3).

In the ABTS assay, ascorbic acid again demonstrated the most potent activity (IC₅₀ = 34.27 ± 1.39 µg/mL). Both ginger juice (31.78 ± 0.63 µg/mL) and lemon juice (33.10 ± 1.74 µg/mL) exhibited substantial antioxidant effects, while the formulation (30.92 ± 2.00 µg/mL) showed slightly reduced efficacy compared to the individual extracts. This result may reflect complex inter-compound interactions that modulate the overall antioxidant response in the blended preparation (Table 3).

The TAC (Total Antioxidant Capacity) assay produced a similar pattern. Ascorbic acid remained the most effective antioxidant (IC₅₀ = 60.14 ± 0.77 µg/mL), while the formulation (63.90 ± 1.11 µg/mL) demonstrated strong activity, exceeding that of lemon juice (59.49 ± 2.34 µg/mL) and ginger juice (58.43 ± 2.20 µg/mL). These closely clustered values suggest a comparable antioxidant potential among the individual extracts and their combination (Table 3).

Further insights were obtained from the FRAP (Ferric Reducing Antioxidant Power) assay. Ascorbic acid once again showed the greatest reducing capacity (IC₅₀ = 75.12 ± 2.24 µg/mL). Among the juices, ginger exhibited the highest reducing power (74.59 ± 1.85 µg/mL), followed by lemon (76.91 ± 2.14 µg/mL). The combined formulation (77.90 ± 1.76 µg/mL) presented slightly lower activity, which could be attributed to potential antagonistic effects among certain phytochemicals (Table 3).

Collectively, these four antioxidant assays confirm the suitability of ascorbic acid as a positive control and establish that both *Zingiber officinale* and *Citrus limon* juices possess significant antioxidant properties. While the combined formulation did not consistently outperform the individual juices, it demonstrated strong and reliable antioxidant activity overall. The observed variability may stem from interactive effects among the compounds in the mixture, which could influence efficacy positively or negatively. Nonetheless, the formulation displays considerable promise for application in nutraceutical and functional food development. Further studies are recommended to elucidate the mechanisms underlying these interactions and to optimize the antioxidant potential of such natural combinations.

**Fig 5:**
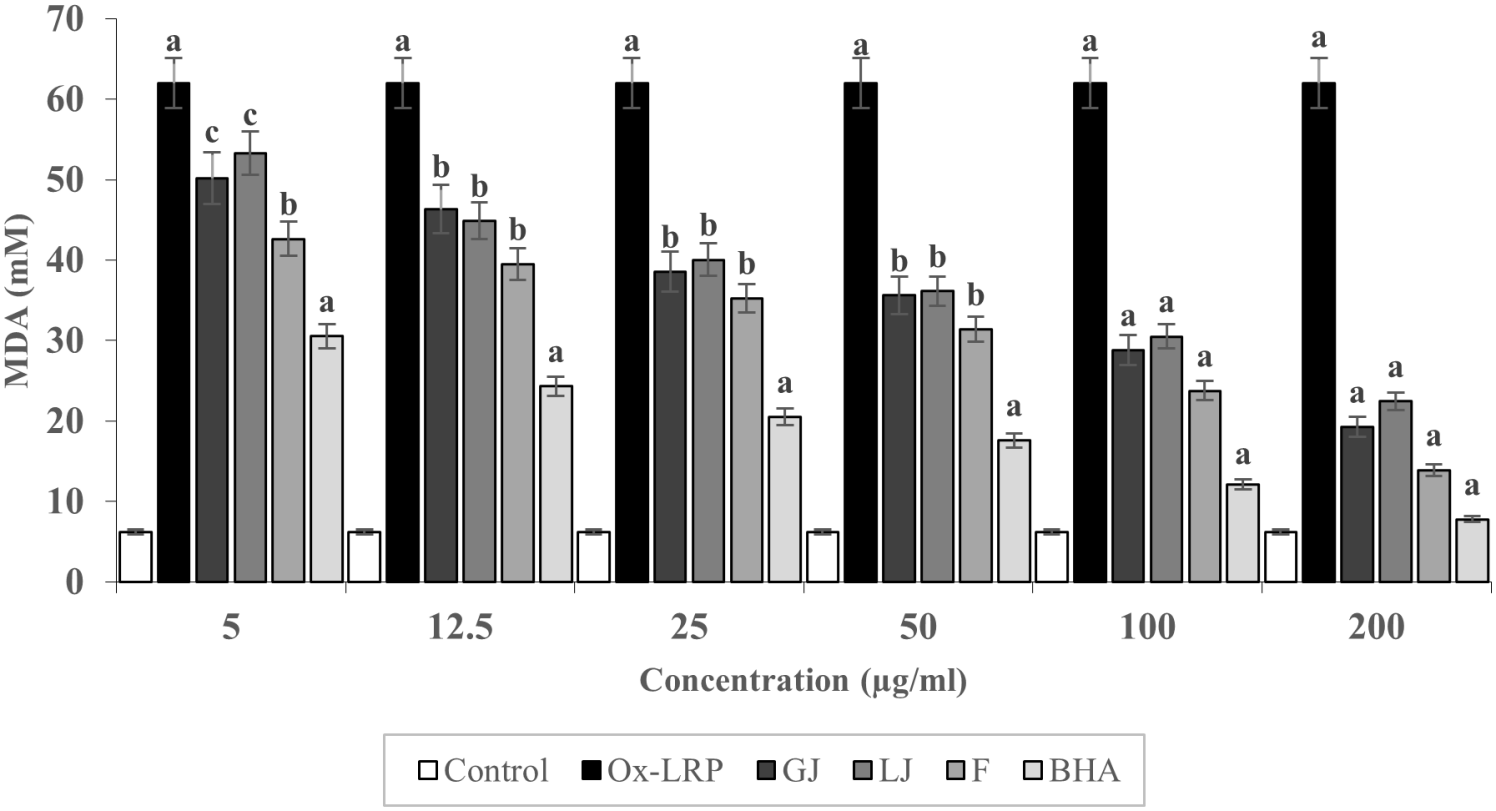
Antioxidant effects of ginger juice (GJ), lemon juice (LJ), and their combination (F) on lipid peroxidation in Triton WR-1339-induced lipid-rich plasma, assessed by TBARS assay. MDA levels are presented as mean ± SEM (n = 6). Statistical significance was determined using one-way ANOVA followed by Tukey’s test. a: p < 0.001, b < 0.01, c: < 0.05 vs. control or Ox-LRP group.

Fig 5 illustrates the antioxidant effects of *Zingiber officinale* juice (GJ), *Citrus limon* juice (LJ), their combined formulation (F), and the synthetic antioxidant butylated hydroxyanisole (BHA), used as a positive control, on lipid-rich plasma (Ox-LRP) subjected to oxidative stress induced by Triton WR-1339. Lipid peroxidation was evaluated using the TBARS assay, with malondialdehyde (MDA) concentrations serving as a biomarker of oxidative lipid damage. The control group, which was not exposed to oxidative stress, displayed low MDA levels, confirming the stability of plasma lipids under physiological conditions. In contrast, the Ox-LRP group showed markedly elevated MDA concentrations across all tested doses, thereby validating the efficacy of the oxidative stress model.

Treatment with GJ resulted in a significant, dose-dependent reduction in MDA levels, highlighting the strong antioxidant activity of *Zingiber officinale*. This effect was especially evident at higher concentrations (100–200 µg/mL), likely due to the presence of bioactive compounds such as gingerols and shogaols, which are known to combat lipid peroxidation. Similarly, LJ demonstrated a consistent antioxidant response, with a notable decrease in MDA levels observed from concentrations as low as 50 µg/mL. The efficacy of LJ is likely attributable to its rich content of flavonoids and polyphenolic compounds, which play critical roles in neutralizing free radicals and preserving lipid membrane integrity (Fig 5).

The combined formulation (F) exhibited even greater antioxidant capacity than the individual juices at equivalent concentrations, suggesting a synergistic interaction between the phytochemicals in ginger and lemon. The formulation’s effect on MDA reduction at higher doses closely approximated that of BHA, indicating that the natural blend can offer protection against lipid peroxidation comparable to that of a potent synthetic antioxidant. This reinforces the therapeutic promise of the GJ–LJ combination as a natural alternative for managing oxidative stress (Fig 5).

The BHA-treated group, serving as a benchmark for antioxidant efficacy, maintained MDA levels near baseline across all tested concentrations, thereby confirming the reliability and sensitivity of the TBARS assay. Statistical differences between experimental groups, indicated by distinct superscript letters, further validate the significance and reproducibility of the results. Collectively, these findings provide strong evidence that the synergistic blend of *Zingiber officinale* and *Citrus limon* offers superior protection against oxidative stress compared to their individual use, supporting its potential development as a natural therapeutic intervention for oxidative damage and related metabolic disorders (Fig 5).

### 3.5. Hypolipidemic effect of *Zingiber officinale* and *Citrus limon* juices on mice

**Fig 6:**
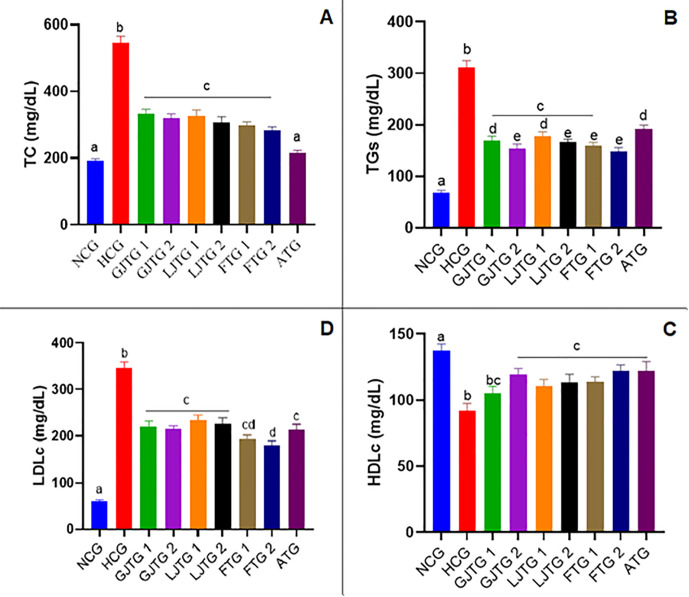
Effects of GJ, LJ, and their formulation (F) on plasma lipid profile in hyperlipidemic mice. Measured parameters include total cholesterol **(A)**, triglycerides **(B)**, HDL-c **(C)**, and LDL-c **(D)**. Data are mean ± SEM (n = 6). Significance tested via one-way ANOVA with Tukey’s post hoc test. c: p < 0.05, b: p < 0.01, a: p < 0.001.

The results of hypolipidemic activity are represented in Fig 6. Triton injection significantly increased triglycerides, low-density lipoprotein (LDLc), and total cholesterol while considerably lowering high-density lipoprotein (HDLc) when compared to average values (p < 0.05) (Fig 6-A to Fig 6-D).

However, all treatments induced significant lipid-lowering effects compared to the hyperlipidemic group (p < 0.05). Indeed, administering juices at all concentrations and their combinations reduced elevated lipids with a comparable impact to atorvastatin (10 mg/kg). Interestingly, at 500 mg/kg, the *Zingiber officinale* and *Citrus limon* juice formulation revealed a greater reduction in TG and LDL (p < 0.05), suggesting a synergistic effect.

### AIP, LDL/HDL, TC/HDL, and TG/HDL ratios calculation

The atherogenic index of plasma (AIP) is a well-established biomarker that reflects the equilibrium between atherogenic lipoproteins, such as LDL-C, and protective HDL-C particles. Elevated AIP values are strongly associated with increased cardiovascular risk, as they signify a greater likelihood of lipid accumulation within arterial walls and a propensity toward atherogenesis. In the normolipidemic control group (NCG), the AIP remains low, around 0.43, indicative of a favorable lipid profile and minimal cardiovascular threat (Table 4). This profile suggests efficient reverse cholesterol transport by HDL-C, maintaining vascular homeostasis.

**Table 4:** Effects of *Zingiber officinale* and *Citrus limon* juices on lipid ratios and Atherogenic Index of Plasma in hyperlipidemic mouse models.

Group	AIP	LDL/HDL	TC/HDL	TG/HDL
**NCG**	0.428 ± 0.02	0.642 ± 0.01	1.428 ± 0.05	0.714 ± 0.03
**HCG**	5.018 ± 0.10^a^	3.202 ± 0.15^a^	6.011 ± 0.20^a^	3.005 ± 0.10^a^
**GJTG1**	3.090 ± 0.08^b^	2.025 ± 0.12^c^	4.090 ± 0.15^b^	1.818 ± 0.08^a^
**GJTG2**	2.333 ± 0.07^a^	1.666 ± 0.10^a^	3.333 ± 0.12^a^	1.529 ± 0.07^a^
**LJTG1**	2.565 ± 0.06^a^	1.826 ± 0.09^b^	3.565 ± 0.10^b^	1.652 ± 0.06^a^
**LJTG2**	2.253 ± 0.05^a^	1.666 ± 0.08^a^	3.256 ± 0.09^a^	1.538 ± 0.05^a^
**FTG1**	2.041 ± 0.04^a^	1.524 ± 0.07^a^	3.041 ± 0.08^a^	1.366 ± 0.04^a^
**FTG2**	1.846 ± 0.03^a^	1.384 ± 0.06^a^	2.846 ± 0.07^a^	1.230 ± 0.03^a^
**ATG**	1.592 ± 0.02^a^	1.259 ± 0.05^a^	2.592 ± 0.06^a^	1.111 ± 0.02^a^

In stark contrast, the hyperlipidemic control group (HCG) exhibits a dramatic surge in AIP to approximately 5.00. This pronounced elevation reflects a pathological lipid profile marked by a significant reduction in HDL-C and a rise in total cholesterol levels, attributable to the dysregulation induced by Triton WR-1339 (Table 4). Such a profile denotes a pro-atherogenic state that significantly elevates cardiovascular risk.

Treatment with the tested interventions resulted in a dose-dependent decline in AIP values, signifying their lipid-normalizing effects. For instance, the GJTG1 group achieved a reduction to 3.09, while the FTG2 group reached 2.33. Notably, the atorvastatin-treated group (ATG) displayed the most substantial improvement, with the AIP dropping to 1.59. This reduction suggests that atorvastatin either elevates HDL-C levels or effectively lowers total cholesterol, reestablishing lipid equilibrium. These findings collectively support the therapeutic efficacy of both natural and synthetic treatments in ameliorating dyslipidemia and reducing cardiovascular risk through AIP modulation (Table 4).

The LDL/HDL ratio, another critical index of lipid balance, reflects the relative abundance of atherogenic LDL-C to anti-atherogenic HDL-C. In the NCG group, the ratio is maintained at approximately 0.64, consistent with a cardioprotective lipid profile. However, this ratio increases markedly to 3.2 in the HCG group, indicating significant lipid imbalance and heightened atherogenic risk. Therapeutic interventions led to substantial improvements, with the ATG group again showing the most marked effect, reducing the ratio to 1.26. These results underscore the treatments’ ability to restore lipid homeostasis by modulating the LDL-C and HDL-C balance in favor of cardiovascular health (Table 4).

Additionally, the total cholesterol to HDL-C (TC/HDL) ratio serves as a robust predictor of cardiovascular outcomes. A ratio below 3.5 is generally associated with low cardiovascular risk. In this study, the NCG group exhibited a ratio of 1.43, affirming optimal cholesterol regulation. Conversely, the HCG group recorded a markedly elevated ratio of 6.00, further confirming the dyslipidemic state induced by Triton WR-1339. Treatment groups demonstrated progressive normalization of this ratio, with ATG again showing the greatest improvement, lowering the value to approximately 2.59. This reduction reflects the compound’s capacity to significantly attenuate the atherogenic burden and restore a healthier lipid profile (Table 4).

The triglyceride-to-HDL cholesterol (TG/HDL-C) ratio is increasingly recognized as a sensitive marker for assessing metabolic dysfunction, particularly in relation to insulin resistance and cardiovascular risk. Elevated values typically reflect impaired lipid metabolism and are strongly associated with an increased likelihood of atherogenic dyslipidemia. In the normolipidemic control group (NCG), the TG/HDL-C ratio is approximately 0.71, consistent with a metabolically healthy state and balanced lipid profile (Table 4).

Conversely, the hyperlipidemic control group (HCG) displays a significant elevation in this ratio, reaching 3.00, a pattern that underscores the pronounced hypertriglyceridemic effect induced by Triton WR-1339 and its disruption of lipid homeostasis (Table 4). This shift not only reflects a higher burden of circulating triglycerides but also a relative depletion of protective HDL particles, contributing to a heightened cardiovascular risk profile.

Therapeutic interventions markedly attenuated this dysregulation, with all treatment groups exhibiting substantial improvements. Among them, the atorvastatin-treated group (ATG) again showed the most pronounced effect, reducing the TG/HDL-C ratio to 1.11. This notable improvement indicates the compound’s capacity to target both cholesterol and triglyceride abnormalities simultaneously. Such a dual-action effect reinforces the potential of these interventions, whether synthetic or plant-based, as viable strategies for correcting lipid imbalances and mitigating cardiovascular and metabolic complications (Table 4).

### 3.6. Toxicity study of *Zingiber officinale* and *Citrus limon* juices

#### 3.6.1. Acute toxicity study.

The acute oral toxicity assessment demonstrated that all treated mice maintained normal behavior and physical appearance throughout the 14-day observation period, even at the highest tested dose of 10 g/kg body weight. No signs of toxicity were recorded, including alterations in grooming, locomotor activity, posture, feeding behavior, or body weight. Importantly, no mortality or adverse clinical symptoms were observed during the study.

This absence of observable toxicological effects, even at such a high dose, suggests a broad margin of safety for ginger juice (GJ), lemon juice (LJ), and their combined formulation (F) under the experimental conditions employed. These findings strongly support the non-toxic nature of the tested preparations and reinforce their potential for further development as safe, natural therapeutic agents.

#### 3.6.2. Subacute toxicity study.

a) **Body weight**

**Fig 7:**
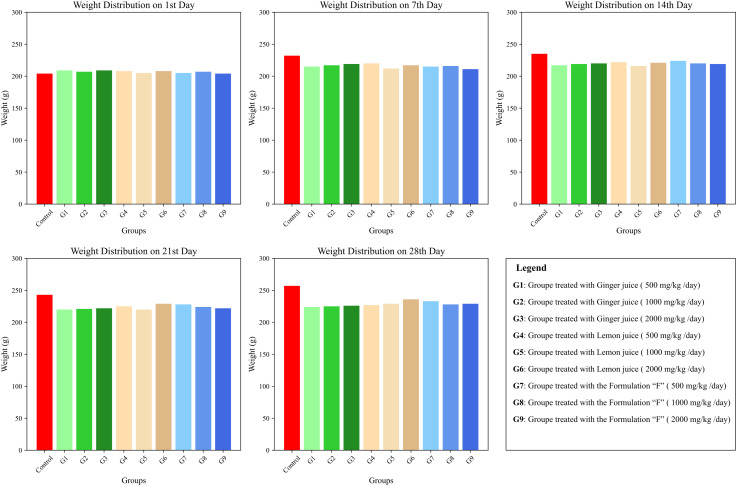
Body Weight variations in rats treated with ginger juice, lemon juice, and their formulation during a 28-day subacute toxicity study. Data are expressed as mean values.

Fig 7 presents the graph illustrating the findings of a 28-day subacute toxicity study, which evaluated the effects of *Zingiber officinale* (ginger juice), *Citrus limon* (lemon juice), and their combined formulation on the body weights of rats. The experiment included a Control and ten treated groups (G1 to G9). The Control group likely received no treatment or a placebo, while the treated groups received varying doses or formulations of the juices. Body weight was regularly measured on the 1^st^, 7^th^, 14^th^, 21^st^, and 28^th^ days to monitor growth patterns and detect potential toxic effects. The rats were housed under standard laboratory conditions, including controlled temperature, proper nutrition, and access to water, to minimize environmental interference with the results.

From the graph, the body weights of all groups, including the Control and treated groups, show a general upward trend throughout the 28-day study period.

At baseline (day 1), body weights were comparable across all experimental groups, indicating successful randomization and uniform starting conditions prior to treatment administration. By day 7, a modest increase in body weight was observed in all groups, including the control and treatment groups (G1 to G9). Notably, the control group exhibited a slightly greater weight gain than the treated groups, as illustrated in Fig 7.

Progressing to Days 14, 21, and 28, body weight continued to increase across all groups; however, the treated groups consistently showed slightly lower gains compared to the control. This difference was most pronounced on Days 21 and 28. Nevertheless, the overlapping error bars suggest that these variations were not statistically significant (Fig 7).

The consistent upward trend in body weight throughout the 28-day period, without any evidence of stagnation or decline, indicates the absence of treatment-related systemic toxicity. This pattern supports the conclusion that the administration of *Zingiber officinale*, *Citrus limon*, and their combination did not produce acute or severe toxic effects.

Since body weight is a widely recognized indicator of systemic well-being in toxicity studies, the continuous weight gain observed across all groups reinforces the safety profile of the tested formulations. The slightly attenuated weight increase in treated animals may reflect mild effects on appetite, metabolism, or nutrient assimilation, an interpretation consistent with the known physiological actions of bioactive constituents such as gingerols, citric acid, and flavonoids. Importantly, the absence of weight loss or growth suppression confirms that the administered doses were well-tolerated and did not impair normal physiological development (Fig 7).

b) **Relative organ weights**

Table 5 presents the absolute and relative organ weight data for rats receiving oral administration of ginger juice (GJ), lemon juice (LJ), and their combined formulation (F) across ten experimental groups. The absolute liver weight in the control group (G1) was recorded at 7.79 ± 0.51 g, with similar values observed among all treatment groups. The highest liver weight was noted in G3 (8.05 ± 0.49 g), while the lowest appeared in G6 (7.85 ± 0.63 g) (Table 5). These findings reflect a general uniformity in liver weights across groups, indicating no significant treatment-related alterations when compared to the control.

**Table 5:** Absolute and relative organ weights of rats administered orally with the GJ, LJ, and their formulation F.

	Parameters	Control	G1	G2	G3	G4	G5	G6	G7	G8	G9
**Liver (g)**	**Absolute weight (g)**	7.79 ± 0.51	7.88 ± 0.40	8.05 ± 0.49	7.96 ± 0.63	7.99 ± 0.63	7.85 ± 0.63	7.91 ± 0.63	8.11 ± 0.63	7.76 ± 0.63	8.02 ± 0.50
	**Relative weight (g)**	3.31 ± 0.19	3.44 ± 0.22	3.40 ± 0.20	3.49 ± 0.28	3.46 ± 0.21	3.38 ± 0.18	3.45 ± 0.27	3.44 ± 0.28	3.40 ± 0.25	3.51 ± 0.26
**Kidney (g)**	**Absolute weight (g)**	0.70 ± 0.01	0.68 ± 0.02	0.65 ± 0.01	0.66 ± 0.02	0.63 ± 0.02	0.61 ± 0.01	0.64 ± 0.02	0.70 ± 0.02	0.66 ± 0.01	0.69 ± 0.02
	**Relative weight (g)**	0.25 ± 0.01	0.27 ± 0.01	0.25 ± 0.01	0.26 ± 0.01	0.27 ± 0.01	0.25 ± 0.01	0.26 ± 0.01	0.28 ± 0.01	0.24 ± 0.01	0.29 ± 0.01

Data are presented as Mean ± SEM; **G1**: control group treated with distilled water; **G2**: group treated with 500 mg/kg/day of GJ; **G3**: group treated with 1000 mg/kg/day of GJ; **G4**: group treated with 2000 mg/kg/day of GJ; **G5**: group treated with 500 mg/kg/day of LJ; **G6**: group treated with 1000 mg/kg/day of LJ; **G7**: group treated with 2000 mg/kg/day of LJ; **G8**: group treated with 500 mg/kg/day of F; **G9**: group treated with 1000 mg/kg/day of F; **G10**: group treated with 2000 mg/kg/day of F.

Relative liver weights followed a pattern like that of absolute weights, with the control group registering a value of 3.31 ± 0.19 g. Slight increases were observed in groups G2 and G9, reaching 3.40 ± 0.20 g and 3.51 ± 0.26 g, respectively, while group G5 showed a comparatively lower value of 3.38 ± 0.18 g. These variations remained minimal and within a narrow physiological range, suggesting that the treatments had no substantial impact on liver mass relative to body weight (Table 5).

For kidney weights, the control group exhibited an absolute value of 0.70 ± 0.01 g. Across the treatment groups, values ranged from 0.61 ± 0.01 g in G5 to 0.70 ± 0.02 g in G7. Slight reductions were noted in G6 (0.64 ± 0.02 g) and G8 (0.66 ± 0.01 g), though these changes were marginal and unlikely to reflect any biologically significant effect. Relative kidney weights were similarly consistent across all groups, with the control group recording 0.25 ± 0.01 g and treated groups ranging from 0.24 ± 0.01 g to 0.29 ± 0.01 g. The highest relative kidney weight was observed in G9 (0.29 ± 0.01 g), corresponding to the highest dose of the combined formulation, yet the increase remained modest and within normal limits (Table 5).

Overall, the comparative analysis of organ weights among the experimental groups reveals that the treatments had minimal impact on both absolute and relative liver and kidney weights. Administration of ginger juice (GJ), lemon juice (LJ), and their combined formulation (F), even at the highest tested doses, did not produce any notable deviations from the control group. This consistency suggests a high level of tolerability. The preservation of stable organ weights across all groups further supports the conclusion that the tested interventions did not induce hepatic or renal toxicity under the experimental conditions employed in this study.

c) **Hematological analysis**

Table 6 presents the hematological profiles of rats subjected to subacute oral administration of ginger juice (GJ), lemon juice (LJ), and their combined formulation (F), across ten experimental groups (G1 to G9). All values are reported as means ± SEM (Table 6)

**Table 6:** Impact of subacute oral administration of GJ, LJ, and their formulation F on the hematological profile of rats.

	WBC (10^9^/L)	RBC (10^12^/L)	HGB (g/dL)	HCT (%)	MCV (fL)	MCH (pg)	MCHC (g/dL)	PLT (10^9^/L)	MPV (fL)
**Control**	4.95 ± 0.13	6.99 ± 0.31	12.96 ± 0.49	38.06 ± 0.42	54.15 ± 0.74	17.96 ± 0.60	35.09 ± 0.57	602.66 ± 17.83	6.49 ± 0.22
**G1**	5.12 ± 0.16	7.17 ± 0.34	13.09 ± 0.46	38.11 ± 0.46	54.18 ± 0.72	18.03 ± 0.58	34.91 ± 0.59	598.17 ± 21.58	6.52 ± 0.20
**G2**	4.89 ± 0.19	7.11 ± 0.32	13.11 ± 0.51	37.96 ± 0.35	53.94 ± 0.70	18.11 ± 0.49	35.07 ± 0.51	611.09 ± 20.99	6.53 ± 0.22
**G3**	4.91 ± 0.17	6.95 ± 0.38	13.12 ± 0.54	38.13 ± 0.33	54.07 ± 0.73	18.14 ± 0.61	35.15 ± 0.56	609.28 ± 24.15	6.44 ± 0.26
**G4**	5.03 ± 0.13	6.85 ± 0.35	12.93 ± 0.46	37.98 ± 0.29	53.93 ± 0.68	18.12 ± 0.53	35.12 ± 0.53	599.53 ± 20.47	6.45 ± 0.31
**G5**	4.97 ± 0.15	7.20 ± 0.37	13.06 ± 0.48	37.90 ± 0.50	54.12 ± 0.74	17.95 ± 0.48	35.26 ± 0.48	608.87 ± 22.70	6.39 ± 0.31
**G6**	4.90 ± 0.19	7.15 ± 0.42	12.98 ± 0.59	37.92 ± 0.34	53.91 ± 0.71	18.13 ± 0.64	34.98 ± 0.52	596.38 ± 19.87	6.48 ± 0.25
**G7**	5.13 ± 0.13	7.14 ± 0.36	13.15 ± 0.61	38.25 ± 0.48	54.03 ± 0.68	17.90 ± 0.57	35.18 ± 0.51	598.05 ± 25.04	6.43 ± 0.29
**G8**	4.99 ± 0.12	6.92 ± 0.43	13.18 ± 0.50	37.97 ± 0.47	54.01 ± 0.70	18.05 ± 0.59	35.12 ± 0.44	604.54 ± 16.08	6.56 ± 0.27
**G9**	4.76 ± 0.14	6.89 ± 0.40	12.99 ± 0.45	38.17 ± 0.31	53.98 ± 0.71	17.98 ± 0.62	35.17 ± 0.50	602.21 ± 23.65	6.51 ± 0.30

Values are expressed as Mean ± SEM; **G1**: group treated with 500 mg/kg/day of GJ; **G2**: group treated with 1000 mg/kg/day of GJ; **G3**: group treated with 2000 mg/kg/day of GJ; **G4**: group treated with 500 mg/kg/day of LJ; **G5**: group treated with 1000 mg/kg/day of LJ; **G6**: group treated with 2000 mg/kg/day of LJ; **G7**: group treated with 500 mg/kg/day of F; **G8**: group treated with 1000 mg/kg/day of F; **G9**: group treated with 2000 mg/kg/day of GJ; WBC: white blood cell count; RBC: red blood cell count; HGB: hemoglobin concentration; HCT: hematocrit; MCV: mean corpuscular volume; MCH: mean corpuscular hemoglobin; MCHC: mean corpuscular hemoglobin concentration; PLT: platelet levels; MPV: mean platelet volume.

White blood cell (WBC) counts exhibited minor intergroup variation, ranging from 4.76 ± 0.14 × 10⁹/L in G9 to 5.13 ± 0.13 × 10⁹/L in G7, closely aligning with the control value of 4.95 ± 0.13 × 10⁹/L. These findings indicate that the treatments had a minimal impact on leukocyte levels. Similarly, red blood cell (RBC) counts remained stable across groups, with values ranging from 6.85 ± 0.35 × 10¹²/L in G4 to 7.20 ± 0.37 × 10¹²/L in G5. The control group recorded an RBC count of 6.99 ± 0.31 × 10¹²/L, and treated groups, including those receiving the combined formulation, remained within this physiological range (Table 6).

Hemoglobin (HGB) levels also demonstrated slight variation, with the highest concentration observed in G8 (13.18 ± 0.50 g/dL) and the lowest in G4 (12.93 ± 0.46 g/dL), compared to 12.96 ± 0.49 g/dL in the control group. Hematocrit (HCT) values followed a similar trend, ranging from 37.96 ± 0.35% to 38.25 ± 0.48% across treatment groups, with the control group at 38.06 ± 0.42%, confirming consistency across all conditions (Table 6).

Erythrocyte indices, including mean corpuscular volume (MCV), mean corpuscular hemoglobin (MCH), and mean corpuscular hemoglobin concentration (MCHC), remain consistent throughout. MCV values ranged narrowly from 53.91 ± 0.68 fL (G6) to 54.18 ± 0.72 fL (G5), while MCH values varied between 17.95 ± 0.48 pg (G5) and 18.11 ± 0.49 pg (G2). MCHC values fluctuated slightly from 34.91 ± 0.59 g/dL in G1 to 35.26 ± 0.48 g/dL in G5, aligning closely with the control group’s value of 35.09 ± 0.57 g/dL (Table 6).

Platelet (PLT) counts were similarly stable, with values ranging from 596.38 ± 19.87 × 10⁹/L in G6 to 608.87 ± 22.70 × 10⁹/L in G5. The control group showed a platelet count of 602.66 ± 17.83 × 10⁹/L. Groups treated with the combined formulation, including G7 (604.54 ± 16.08 × 10⁹/L) and G10 (602.21 ± 23.65 × 10⁹/L), displayed no significant deviations. Mean platelet volume (MPV) values remained within a narrow range across all groups, from 6.39 ± 0.31 fL (G5) to 6.56 ± 0.27 fL (G8), with the control group at 6.49 ± 0.22 fL (Table 6).

In summary, all measured hematological parameters remained within physiological norms, with only minimal variation between groups. These results indicate that oral administration of GJ, LJ, and their combined formulation did not adversely affect blood cell counts, hemoglobin levels, or platelet indices, thus supporting the hematological safety of these treatments.

### 3.7. Molecular docking study

Molecular priming is a highly effective mechanism, providing insights into potential interactions between a molecule and its targeted protein receptor, inferred from the energy discharge during molecular engagements (25–27). This technique is a foundational computational tool frequently used to delineate the critical molecular interactions of pharmacologically active entities.

**Fig 8:**
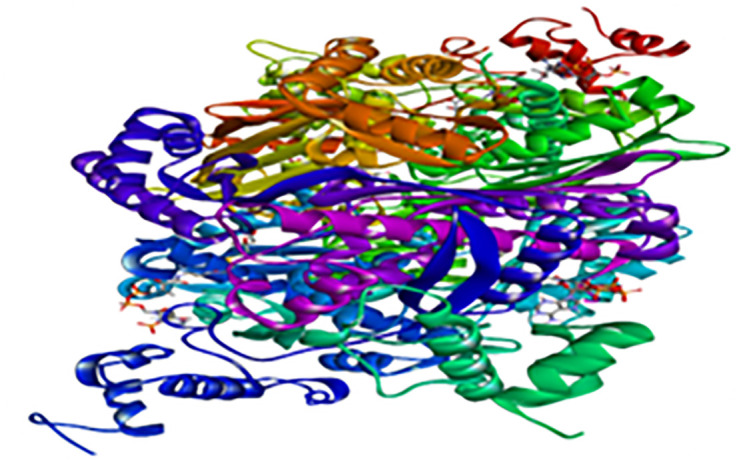
3D structure of the HMG-CoA reductase.

Fig 8 illustrates the structure of one of the primary enzymes involved in lipid metabolism, HMG-CoA reductase.

Additionally, it can be perceived as a preliminary step in the developmental protocol for novel therapeutic agents. The present study conducted priming analyses on four molecules sourced from *C. limon* juice and three from *Z. officinale* juice (Table 3) in conjunction with the HMG-CoA reductase enzyme (Fig 8).

As shown in Table 6, binding energies varied depending on the compound under investigation. Among all ligands assessed, 6-gingerol exhibited the lowest binding affinity for HMG-CoA reductase (Fig 8, Table 6), whereas hesperidin demonstrated the highest. Notably, the binding energy of hesperidin exceeded that of the reference inhibitor for HMG-CoA reductase, indicating a strong likelihood of inhibitory activity against this enzyme and suggesting potential efficacy in reducing endogenous cholesterol synthesis.

**Fig 9:**
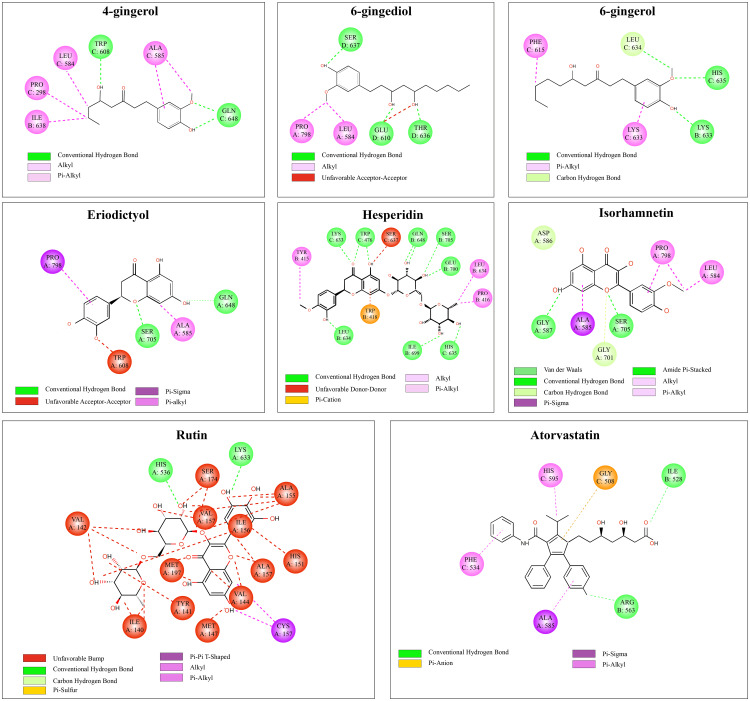
Possible interaction sites of *Zingiber officinale*, *Citrus limon*, and the atorvastatin molecules.

The high affinity of hesperidin is attributed to its robust interactions within the enzyme’s active site. Specifically, hydrogen bonds were formed between the carbonyl oxygen and residues LYS C:633 and HIS C:536. Additional hydrogen bonding occurred between hydroxyl oxygen atoms and several key amino acids, including LEU B:634, ILE B:699, SER C:637, GLU B:700, SER B:705, GLN B:648, HIS C:635, and again LYS C:633 (Fig 9). These interactions were further stabilized by non-covalent forces such as alkyl, π-alkyl, and π-cation interactions, all contributing to hesperidin’s strong binding profile, as illustrated in Fig 9.

For instance, rutin docked into the HMG-CoA reductase active pocket, forming hydrogen bonds with Lys^633 and His^536 [34] (key catalytic residues). Hesperidin formed a network of hydrogen bonds and hydrophobic contacts across the active-site cleft [35], anchoring it in the catalytic site. These binding modes suggest that the phytochemicals occupy the enzyme’s active site similarly to atorvastatin, thereby potentially blocking HMGCR activity [36].

Table 7 provides a comparative overview of the binding energies of several natural compounds isolated from *Zingiber officinale* and *Citrus limon*, along with the reference drug Atorvastatin, against the enzyme HMG-CoA reductase, a key regulator in cholesterol biosynthesis and a well-established pharmacological target in lipid-lowering therapies. This analysis enables the evaluation of each compound’s inhibitory potential based on binding affinity, where more negative values reflect stronger molecular interactions and a higher likelihood of effective enzyme inhibition.

**Table 7:** Binding energy values of natural compounds from ginger and lemon vs. HMG-CoA reductase, compared with Atorvastatin. Lower binding energy indicates stronger predicted interaction affinity (based on molecular docking).

Molecule	Binding Energy (Kcal/mol)
Rutin	−9
Hesperidin	−10.4
Eriodictyol	−8.5
Isorhamnetin	−8
6-gingerol	−5.5
6-gingediol	−6.1
4-gingerol	−6.1
Atorvastatin	−8.1

Among the compounds tested, hesperidin displayed the strongest interaction with HMG-CoA reductase, achieving a binding energy of −10.4 kcal/mol, surpassing that of Atorvastatin (−8.1 kcal/mol). This notable finding positions hesperidin as a compelling natural candidate with significant potential for modulating lipid levels. Similarly, rutin (−9.0 kcal/mol) and eriodictyol (−8.5 kcal/mol) exhibited strong binding affinities, suggesting their relevance for further pharmacological exploration. Isorhamnetin, with a slightly lower affinity (−8.0 kcal/mol), also warrants consideration for its therapeutic potential (Table 7).

In contrast, ginger-derived compounds such as 6-gingerol (−5.5 kcal/mol), 6-gingediol (−6.1 kcal/mol), and 4-gingerol (−6.1 kcal/mol) showed more moderate binding energies, indicating comparatively weaker interactions with the enzyme (Table 7). These differences suggest that while ginger constituents may not act as primary inhibitors, they could play a supportive or complementary role within broader lipid-regulating mechanisms.

The comparison with Atorvastatin underscores the pharmacological relevance of certain natural compounds, particularly hesperidin and rutin, which demonstrated superior binding affinities. These findings highlight the potential of such molecules to serve as safer, more tolerable alternatives to conventional statins, especially considering the lower incidence of adverse effects typically associated with plant-based therapies. Nonetheless, these *in silico* observations require further validation through *in vitro* and *in vivo* studies to confirm their biological activity and therapeutic viability.

From a clinical perspective, these results have important implications. Natural inhibitors of HMG-CoA reductase may contribute to cholesterol regulation and cardiovascular protection. While the relatively lower affinities observed with ginger-derived compounds point to limited direct inhibition, they may exert their benefits through alternative pathways, supporting the rationale for exploring combination-based therapeutic strategies.

In summary, the data presented in Table 8 underscore the promising role of phytochemicals from *Citrus limon* and *Zingiber officinale* in modulating cholesterol biosynthesis via HMG-CoA reductase inhibition. Hesperidin, in particular, emerged as the most potent candidate, exhibiting greater binding affinity than the reference drug Atorvastatin. These insights reinforce the growing interest in plant-derived compounds as effective and well-tolerated agents for managing hyperlipidemia and pave the way for future research into natural product-based therapeutic approaches aimed at improving cardiovascular health.

**Table 8:** Summary of Key Findings.

Parameter	GJ	LJ	Formulation (GJ + LJ)
**Total Phenolic Content**	High phenolic content	Moderate phenolic content	Synergistically enhanced
**Total Flavonoid Content**	High flavonoid content	High flavonoid content	Synergistically enhanced
**HPLC**	[4]-gingerol, [6]-gingerol, and [6]-gingediol	Eriodictyol, hesperidin, rutin, and isorhamnetin	Combined profile
**Antioxidant Activity (DPPH, ABTS, TAC, FRAP)**	Strong capacity (low IC_50_)	Moderate capacity	Synergistic effect
**TBARS (MDA levels)**	↓ MDA levels	↓ MDA levels	↓↓ MDA levels
**Total Cholesterol**	↓ vs. Triton	↓ vs. Triton	↓↓ vs. Triton
**Triglycerides**	↓ vs. Triton	↓ vs. Triton	↓↓ vs. Triton
**HDL-c**	↑	↑	↑↑
**LDL-c**	↓	↓	↓↓
**Atherogenic Index**	Improved	Improved	Significantly improved
**Acute Toxicity (single dose)**	No mortality at 2 g/kg	No mortality at 2 g/kg	No mortality at 2 g/kg
**Subacute Toxicity (28 days)**	No significant toxicity observed	No significant toxicity observed	No signs of toxicity, normal weight gain
**Organ Weights**	No significant changes	No significant changes	No organ damage observed
**Hematological Parameters**	Within normal ranges	Within normal ranges	No hematological abnormalities
**Molecular Docking (HMG-CoA Reductase)**	Moderate binding affinity	Moderate binding affinity	High binding affinity (close to Atorvastatin)

## 4. Discussion

Regardless of the underlying causes, the global prevalence of hyperlipidemia is on the rise, along with the healthcare costs associated with this condition. This disorder is known to precipitate serious complications such as atherosclerosis, cardiovascular and cerebrovascular infarctions, heart disease, and stroke [37]. In fact, elevated lipid levels can adversely alter the lipid profile and significantly increase the risk of cardiovascular disease, contributing to at least 2.8 million adult deaths each year. [38]. Considering these consequences, the development of novel lipid-lowering agents with improved safety and efficacy has become especially important. For experimental purposes, acute hyperlipidemia is often induced in animal models using Triton WR-1339, a nonionic detergent (tertiary polymer octylphenol-oxyethylated formaldehyde), as a means to evaluate potential anti-hyperlipidemic compounds (both natural and synthetic) [29] and to investigate cholesterol and triacylglycerol metabolism [39]. One proposed mechanism behind Triton WR-1339’s action is the inhibition of lipoprotein lipase activity, which leads to an accumulation of lipids in plasma [28].

Approximately 24 hours after a Triton injection, plasma lipid levels can surge to roughly two to three times higher than baseline, followed by a gradual decline over the next day. The Triton-induced hyperlipidemia model thus provides a relatively quick and straightforward approach to test candidate anti-hyperlipidemic drugs, serving as a convenient supplementary screening tool in early-phase evaluations [40]. Thus, it is used in numerous scientific investigations into hyperlipidemia research, using different animal models, such as rats [41], mice [42], hamsters [43], and guinea pigs [44].

Since ancient times, medicinal and aromatic plants have been used to treat several disorders, including cardiovascular disease [45]. In recent years, accumulating clinical and experimental evidence has reinforced the therapeutic value of many herbal preparations in promoting lipid regulation and reducing cardiovascular risk. [46–48]. The use of phytoconstituents and natural remedies in the management of hyperlipidemia and heart disease offers a promising alternative, particularly due to their safety and the lower incidence of adverse effects compared to synthetic lipid-lowering drugs [48,49]. Based on this overview, we aim to evaluate the anti-hyperlipidemic activity of the *Zingiber officinale* and *Citrus limon* juice.

In this context, we designed the present study to evaluate the anti-hyperlipidemic potential of *Zingiber officinale* and *Citrus limon* juices. This approach offers three main strengths. First, we assessed the lipid-lowering effects of each juice independently as well as in combination (using equal proportions of both juices). Second, we used fresh juices that are readily accessible and safe to administer, rather than extracts obtained through organic solvents. Third, we complemented our *in vivo* findings with a supporting *in silico* analysis conducted using “AutoDock Vina.software”.

The “LJ” extract exhibited high levels of polyphenols and flavonoids, with total polyphenols measured at 25.23 ± 1.54 mg GAE per g of extract and total flavonoids at 12.75 ± 3.08 mg QE per g. This extract was particularly rich in compounds such as eriodictyol, rutin, hesperidin, and isorhamnetin. In contrast, the “GJ” extract contained lower yet significant quantities of these phytochemicals, with total polyphenols of 18.48 ± 1.14 mg GAE/g and flavonoids of 7.26 ± 2.05 mg QE/g, respectively. Phytochemical profiling confirmed the presence of 4-gingerol, 6-gingediol, and 6-gingerol in the GJ extract. These results align with those of a previous study [50], which also confirmed that *Zingiber officinale*, *Citrus limon*, and their combined formulation contain a wide array of phenolic and flavonoid compounds.

The antioxidant assays performed in this study confirmed that *Zingiber officinale* and *Citrus limon* juices, as well as their combined formulation, possess significant antioxidant activity. This finding is consistent with previous research on the antioxidant properties of these botanicals, while also extending that knowledge. Moreover, the IC_50_ values we obtained for *Zingiber officinale* and *Citrus limon* juices in both DPPH and ABTS assays are in close agreement with earlier reports. For example, Pacula et al. observed DPPH assay IC_50_ values of about 35–45 µg/mL for ginger extracts, which closely match our results [51]. This finding closely mirrors the IC_50_ value of 38.91 ± 1.47 µg/mL observed in the present study. Similarly, the IC_50_ values recorded for lemon juice are in line with those reported by Kumar et al, [52], where *Citrus limon* extracts demonstrated IC_50_ values ranging from 40 to 50 µg/mL, depending on the extraction solvent. These similarities suggest that the antioxidant efficacy of these plant extracts remains consistently robust across different experimental protocols and methodological variations.

Interestingly, the combination of ginger and lemon juices did not consistently yield enhanced antioxidant activity compared to the individual extracts. This observation contrasts with the findings of Zhang et al. [53], who reported a synergistic enhancement in antioxidant activity when ginger and citrus extracts were combined. It is plausible that variations in extract ratios and in the concentrations of bioactive constituents, such as phenolic and flavonoid compounds, could modulate overall antioxidant efficacy. Further investigations examining optimal blending proportions and the specific contributions of individual phytochemicals are needed to clarify these interactions.

The results from the TAC and FRAP assays revealed relatively uniform antioxidant capacities across all samples. These findings align with those of Antoniewicz et al. [54], who highlighted the substantial ferric-reducing power and total antioxidant potential of *Zingiber officinale* and *Citrus limon* when tested separately. However, the slight decrease in FRAP activity observed in their combined formulation suggests the possibility of antagonistic interactions among certain phytochemicals. Such phenomena have indeed been documented by Chen et al., who noted that polyphenol-rich plant combinations do not always yield synergistic effects [55], who noted that polyphenol-rich plant combinations do not always produce synergistic effects.

Ascorbic acid, used as a positive control in all assays, consistently exhibited superior antioxidant activity compared to the natural extracts. This finding corroborates the well-established antioxidant properties of ascorbic acid, including its role as an effective radical scavenger and reducing agent, as described by Kaur and Kapoor [56]. Nevertheless, the relatively close IC_50_ values of the natural extracts to ascorbic acid in the TAC and FRAP assays highlight their potential as alternative sources of antioxidants, particularly in functional food applications.

Additionally, the variation in IC_50_ values between the DPPH and ABTS assays is noteworthy. This divergence aligns with previous literature, which highlights the mechanistic differences between the two methods: DPPH primarily assesses hydrogen atom donation, whereas ABTS captures both hydrogen atom and electron transfer mechanisms. These methodological differences likely account for the slight discrepancies observed in antioxidant rankings. The antioxidant potential of *Zingiber officinale* (GJ), *Citrus limon* (LJ), and their combined formulation (F) was further validated by their ability to inhibit lipid peroxidation, as evidenced by significant reductions in malondialdehyde (MDA) levels in the TBARS assay. A lipid-rich plasma model induced by Triton WR-1339 effectively simulated oxidative stress, thereby enabling the evaluation of the extracts’ protective effects. GJ exhibited a pronounced dose-dependent reduction in MDA levels, likely attributable to the presence of 4-gingerol, 6-gingerol, and 6-gingediol, compounds known for their radical-scavenging activity and anti-inflammatory properties. Similarly, LJ demonstrated potent antioxidant activity at higher concentrations, attributable to its rich flavonoid profile (notably including hesperidin, eriodictyol, isorhamnetin, and rutin). These molecules are recognized for their capacity to stabilize cellular membranes, chelate transition metal ions, and inhibit lipid peroxidation. The combined formulation (F) showed superior efficacy compared to the individual extracts, particularly at higher doses, suggesting a synergistic interaction. This enhanced activity may stem from complementary actions of hydrophilic antioxidants (such as hesperidin) and lipophilic constituents (including 6-gingediol), which together enable a dual-phase (aqueous and lipid) protective effect against oxidative damage. Our results are consistent with prior studies that underscore the antioxidant potential of *Zingiber officinale* and *Citrus limon*. For example, Bekkouch et al. (2022) demonstrated that ginger and lemon juice extracts, rich in bioactive compounds such as 4-gingerol, 6-gingediol, hesperidin, and rutin, exhibit pronounced antioxidant and hepatoprotective effects in a rat model of CCl_4_-induced liver injury [57]. Similarly, research by Köksal and Gülçin (2017) showed that ethanol-based ginger extracts possessed stronger antioxidant capacity than water-based extracts, a finding attributed to their higher concentrations of phenolic acids, notably ferulic and p-coumaric acids [58].

Additional evidence is provided by Shende (2024), who evaluated the biological activity of aqueous and ethanolic extracts of *Citrus medica* and *Zingiber officinale*, reporting significant antioxidant, antibacterial, and anthelmintic properties [59]. Taken together, these findings reinforce the therapeutic potential of these plants and support our observation that their combination can exert enhanced protective effects against oxidative stress. In our study, the antioxidant activity of the formulation nearly matched that of butylated hydroxyanisole (BHA), a widely used synthetic antioxidant, further underscoring its potential as a natural alternative for mitigating oxidative damage. While our results are consistent with the broader literature on polyphenol-rich plant extracts, they also highlight the added value of combining specific phytochemicals. Such synergistic interactions likely contribute to a broader spectrum of protection, providing new insights for the design of effective natural antioxidant formulations. Beyond their antioxidant activity, the present findings reveal that administering *Zingiber officinale* and *Citrus limon* juices produces a lipid-lowering effect. The reduction in plasma lipid levels was comparable to that achieved with Atorvastatin, a standard pharmacological agent used to manage hyperlipidemia. This similarity suggests that these plant juices may confer similar benefits via alternative mechanisms. In the context of lipid regulation, it is worth noting that conventional therapies often target distinct molecular pathways. For instance, fibrates such as fenofibrate act as PPARα agonists, thereby lowering triglyceride levels and raising HDL-C levels. [60]. Bile acid sequestrants and niacin also contribute to improved lipid profiles [61]. More recently, PCSK9 inhibitors, monoclonal antibodies that prevent LDL receptor degradation, have emerged as highly effective agents for reducing LDL-C by over 50% in many cases [62]. Situating the effects of ginger and lemon juices alongside these pharmacologic strategies provides a helpful framework for appreciating their therapeutic potential.

In addition to *Zingiber officinale* and *Citrus limon*, several other plants have demonstrated hypolipidemic properties [43,63]. For example, polyphenol-rich extracts of basil and thyme have been shown to inhibit the synthesis of both triglycerides and cholesterol [42,64].

The improvements in lipid profile observed with the 500 mg/kg dose of the ginger–lemon blend were strikingly similar to those achieved with atorvastatin (10 mg/kg) in our experimental model. Specifically, the decrease in LDL-cholesterol levels resulting from the high-dose natural formulation closely mirrored that seen in the group treated with the statin [65]. This finding is particularly compelling considering that Atorvastatin is a well-established synthetic HMG-CoA reductase inhibitor routinely prescribed for hyperlipidemia management. The data suggest that the combined use of ginger and lemon juices may approximate the lipid-lowering efficacy of this standard pharmacological agent. Moreover, the favorable comparison underscores the therapeutic promise of synergistic plant-based interventions as potential complements or alternatives to conventional dyslipidemia treatments.

Comparable outcomes have been documented for phenolic phytochemicals derived from various medicinal herbs [66–69]. These compounds are known to hinder cholesterol uptake in the intestine, downregulate endogenous cholesterol production, enhance reverse cholesterol transport, and facilitate hepatic excretion of cholesterol via bile. Furthermore, polyphenols have been shown to stimulate LDL receptor expression in the liver, thereby promoting efficient clearance of circulating cholesterol [70]. In addition, polyphenols may activate hepatic low-density lipoprotein (LDL) receptors, facilitating cholesterol clearance in the form of bile acids [71]. They also modulate cholesterol metabolism by regulating key enzymes such as HMG-CoA reductase [72], acyl CoA cholesterol acyl transferase (ACAT) [73], and lecithin cholesterol acyltransferase (LCAT) [46]. The pronounced lipid-lowering effect observed with ginger and lemon juices in our study is likely attributable to their richness in phenolic bioactives, aligning with previously identified mechanisms supported by previous studies [17,74–77].

In the present study, we adopted an innovative extraction approach that employed the juices themselves as the medium for isolating bioactive constituents, thereby eliminating the need for conventional heat- or solvent-based methods. This use of unprocessed juice extracts allowed for the preservation of the natural biological activity of the targeted compounds, while simultaneously avoiding the ecological drawbacks linked to chemical solvent usage. By forgoing traditional extraction techniques, this solvent-free strategy not only expands the methodological repertoire in natural compound isolation but also introduces a sustainable, environmentally conscious pathway for advancing research in natural product chemistry. In this study, treatment with the *Zingiber officinale* and *Citrus limon* juice blend at a dosage of 500 mg/kg resulted in a substantially greater reduction in plasma lipid concentrations than those observed with lower doses or individual treatments. This pronounced lipid-lowering effect appears to stem from a synergistic interaction between the gingerols in *Zingiber officinale* and the flavonols richly present in *Citrus limon*. Notably, the combined formulation produced significant improvements in both LDL-C and HDL-C levels compared to either extract administered alone (p < 0.05), indicating a synergistic rather than merely additive effect. The synergistic effect observed may be attributed to the distinct but complementary biological actions of the phytochemicals present in each plant. Flavonoids derived from citrus, such as hesperidin, have been shown to improve lipid profiles by inhibiting both cholesterol synthesis and intestinal uptake, while simultaneously influencing the expression of key genes that regulate lipid homeostasis [78]. In parallel, constituents such as 6-gingerol found in ginger have been reported to support hepatic clearance of circulating cholesterol by upregulating LDL receptor expression and stimulating pathways involved in cholesterol efflux [79]. The convergence of these mechanisms, diminished cholesterol production and absorption by lemon flavonoids and increased cholesterol removal by gingerols, likely explains the superior hypolipidemic profile observed in the combined treatment group.

Beyond the immediate findings of our study, this synergistic interaction echoes broader themes emerging in botanical polytherapy. For example, a recent investigation [80] demonstrated that a multi-herbal formulation containing *Zingiber officinale*, *Coscinium fenestratum*, and *Carthamus tinctorius* significantly downregulated PCSK9 expression in HepG2 cells, resulting in increased availability of LDL receptors. This dual mechanism, combining inhibition of cholesterol synthesis via HMG-CoA reductase with enhanced LDL clearance through the PCSK9-LDLR pathway, underscores the therapeutic promise of pairing phytochemicals with complementary modes of action. Such integrative approaches may provide a more nuanced strategy for managing hyperlipidemia, potentially reducing dependence on high-dose monotherapies and aligning with a more holistic and sustainable model of care.

A growing body of evidence supports the role of phytochemicals in regulating lipid metabolism and combating hyperlipidemia. Among these, isorhamnetin has garnered significant attention for its ability to mitigate oxidative damage in macrophages exposed to oxidized low-density lipoprotein (ox-LDL) [81]. Mechanistic investigations have demonstrated that this flavonoid mitigates the development of atherosclerotic plaques by inhibiting macrophage apoptosis. This protective effect is mediated through activation of the PI3K/AKT signaling cascade and upregulation of heme oxygenase-1 (HO-1) expression in THP-1-derived macrophages exposed to oxidized LDLs [82]. *In vivo*, isorhamnetin has also demonstrated dose-dependent lipid-lowering properties in rodent models of hyperlipidemia [83].

Constituents derived from ginger have likewise been implicated in the regulation of lipid metabolism. In particular, 6-gingerol has exhibited antihyperlipidemic effects in experimental settings, including models of hyperlipidemia induced by poloxamer P-407 [84]. In models of dyslipidemia induced by a high-fat diet, gingerol was found to enhance lipid profiles while also influencing the expression of several key enzymes involved in lipid metabolism, including lipoprotein lipase, lecithin–cholesterol acyltransferase (LCAT), HMG-CoA reductase, and acetyl-CoA carboxylase. These effects were accompanied by notable anti-inflammatory activity [85].

Beyond their direct lipid-lowering properties, ginger-derived bioactive compounds also influence key transcriptional regulators involved in lipid homeostasis. Zingerone, for example, has been shown to activate peroxisome proliferator-activated receptors (PPARs) while inhibiting NF-κB signaling, thereby contributing to both anti-inflammatory and lipid-regulatory effects [86]. Similarly, 10-gingerol has been shown to increase LDL receptor expression by upregulating sterol regulatory element-binding protein-2 (SREBP-2) and downregulating proprotein convertase subtilisin/kexin type 9 (PCSK9). Additionally, it facilitates cholesterol efflux by activating the LXRα/PPARγ pathways through transporters such as ABCG5/8 and ABCA1 [87]. These observations indicate that the therapeutic benefits of ginger extend beyond the inhibition of HMG-CoA reductase, involving a broader range of regulatory processes at both the transcriptional and post-translational levels. Similarly, hesperidin, a major flavonoid found in lemon, has been shown to modulate LDL receptor expression and impact inflammatory signaling through comparable molecular pathways. [87].

Given that HMG-CoA reductase plays a pivotal role in cholesterol biosynthesis and serves as a key regulatory point in the mevalonate pathway [88], inhibiting its activity remains one of the most effective strategies for managing elevated LDL levels and reducing cardiovascular risk [89]. In our study, molecular docking analyses demonstrated that compounds such as hesperidin, rutin, and eriodictyol displayed more favorable binding affinities to HMG-CoA reductase than atorvastatin, indicating a strong *in silico* potential for inhibitory activity.

To gain deeper insight into this interaction, we analyzed the molecular docking profiles of bioactive compounds derived from *Zingiber officinale* and *Citrus limon* against HMG-CoA reductase, a key enzymatic target commonly associated with cholesterol-lowering therapies [90,91]. The comparative docking results offered valuable insights into the relative inhibitory potential of these molecules compared to the native ligand, thereby reinforcing their possible role in modulating cholesterol levels.

Although HMG-CoA reductase inhibition represents a key pathway in cholesterol regulation, the antioxidant and lipid-lowering effects observed in this study are likely mediated through additional, interconnected molecular mechanisms. One such pathway involves the activation of PPAR-α and PPAR-γ, nuclear receptors that play critical roles in lipid oxidation, glucose homeostasis, and insulin sensitivity. [92]. Furthermore, the anti-inflammatory effects of ginger and lemon may contribute by downregulating pro-inflammatory cytokines such as TNF-α, IL-6, and NF-κB—key mediators implicated in both oxidative stress and dyslipidemia [93]. Collectively, these findings support a multifaceted model in which phytochemical compounds act in concert to regulate both lipid metabolism and inflammatory responses. Further research is warranted to explore these interconnected pathways and to more fully elucidate the therapeutic potential of such botanical interventions.

3-hydroxy-3-methylglutaryl coenzyme A (HMG-CoA) reductase serves as a key regulatory enzyme in cholesterol biosynthesis, functioning as the rate-limiting step within the mevalonate pathway [90,91]. Beyond its fundamental role in maintaining intracellular cholesterol levels, it also represents a pivotal checkpoint in overall cellular metabolic regulation [26,57]. To evaluate whether our natural formulation could effectively target this enzyme, we performed a molecular docking analysis using the crystallographic structure of HMG-CoA reductase (PDB ID: 1DQ9), obtained from the Protein Data Bank.

Our *in silico* evaluation indicated that several bioactive constituents from *Zingiber officinale* and *Citrus limon* extracts exhibited strong binding interactions with HMG-CoA reductase. Notably, hesperidin, rutin, and eriodictyol showed particularly high binding affinities, exceeding that of atorvastatin, the clinically established reference statin. These results suggest that these flavonoids may function as effective inhibitors of HMG-CoA reductase, with the potential to compete with conventional pharmacological agents targeting this enzyme.Rutin, for example, demonstrated a binding energy of −9.0 kcal/mol and established stable hydrogen bonds with key catalytic residues, including LYS C:633 and HIS C:536. Its interaction network also included alkyl, π-alkyl, and π-cation interactions, contributing to the compound’s structural stability within the enzyme’s active site. Hesperidin yielded an even stronger docking score of −10.4 kcal/mol, anchoring itself via multiple hydrogen bonds involving residues from both chains B and C. Its binding profile was further characterized by π-cation and π-alkyl stacking interactions, along with a few donor–donor repulsions suggestive of conformational specificity. Although eriodictyol showed a slightly lower docking score of −8.5 kcal/mol, it engaged the enzyme through several hydrogen bonds and van der Waals forces, supporting a credible and energetically favorable binding mode within the active site.

The cumulative docking findings reinforce the hypothesis that these plant-derived flavonoids are capable of effectively binding to and stabilizing the active site of HMG-CoA reductase. Notably, the binding energies observed for hesperidin, rutin, and eriodictyol were consistently more negative than those of atorvastatin, suggesting a strong potential for high-affinity interaction with the enzyme. These results lend further support to the idea that natural inhibitors could represent viable alternatives or complementary agents to synthetic statins in cholesterol-lowering interventions.

Given the central role of HMG-CoA reductase in regulating cholesterol biosynthesis and its involvement in the development of atherosclerosis and cardiovascular disease [88,94], the strong binding affinities observed *in silico* for these phytochemicals highlight their considerable therapeutic potential. These findings align with our *in vivo* results and further support the relevance of targeting this enzymatic pathway using naturally occurring compounds found in ginger and lemon extracts [89]. While several of the evaluated compounds exhibited promising binding affinities, none surpassed the interaction strength observed with the reference agent, atorvastatin. This outcome highlights the importance of assessing docking results not solely on the basis of binding energy scores, but through a more nuanced interpretation that considers the nature and quality of pharmacodynamic interactions at the molecular level.

In this investigation, molecular docking simulations were purposefully directed toward the catalytic domain of HMG-CoA reductase, with particular attention to the active site identified by the co-crystallized ligand in the 1DQ9 structure. The interactions observed between the candidate compounds and key residues, namely Lys633 and His536, provide compelling evidence for competitive binding, mirroring the mechanism of inhibition typically associated with statins. This aspect is critical in evaluating therapeutic relevance, as both the binding location and interaction type shape the efficacy and pharmacological behavior of a compound. Competitive inhibitors, such as statins, are known to suppress enzymatic activity without provoking unintended downstream effects, whereas non-competitive or allosteric agents may lead to inconsistent potency or off-target consequences. Our findings revealed that hesperidin (−10.4 kcal/mol) and 6-gingerol (−9.6 kcal/mol) engage directly with the active site, lending strong support to the notion that these molecules function as competitive inhibitors. This reinforces their potential pharmacodynamic alignment with existing lipid-lowering drugs.The *in silico* findings presented here are strongly concordant with the lipid-lowering effects documented *in vivo*, thereby strengthening the translational relevance of these compounds as promising candidates for the treatment of hypercholesterolemia.

In addition, recent findings by Ongtanasup and collaborators have highlighted complementary pathways through which these compounds may exert their effects. Specifically, bioactive constituents from *Zingiber officinale* have been shown to downregulate PCSK9 expression, thereby increasing the availability of LDL receptors and facilitating more efficient cholesterol clearance [80]. Additional studies exploring advanced drug delivery strategies, such as ginger-silver nanoparticles [7], and red palm-wax niosomal gels [8] have further enhanced the pharmacological profile of ginger, underscoring the critical role of nanocarrier systems in improving its bioavailability and extending the duration of its therapeutic effects.

Despite their promising pharmacological properties, one of the major challenges in the clinical translation of these phytochemicals lies in their inherently poor systemic bioavailability. Compounds such as 6-gingerol are subject to extensive phase II metabolism in both the intestinal tract and the liver, where they are rapidly converted into glucuronide conjugates. This metabolic transformation significantly reduces their circulating free form and limits their plasma half-life to approximately 1–3 hours [95,96]. Likewise, hesperidin exhibits low water solubility and restricts intestinal absorption. Its conversion into the bioactive form, hesperidin, depends on enzymatic transformation by gut microbiota, adding further complexity to its pharmacokinetic behavior [97,98]. These factors must be taken into account when considering the *in vivo* efficacy of these compounds and in designing dosage strategies for potential clinical applications.

The toxicological assessments performed under both acute and subacute conditions offer important confirmation of the safety profile of the tested extracts. In acute toxicity testing, no behavioral disturbances, clinical signs of distress, or mortality were observed, even at the highest administered doses, suggesting a wide safety margin. Similarly, subacute exposure to *Zingiber officinale*, *Citrus limon*, and their combined preparations did not lead to any significant changes in absolute or relative weights of the liver and kidneys, indicating an absence of target-organ toxicity. In addition, detailed hematological analysis, including red and white blood cell counts, hemoglobin concentration, hematocrit levels, platelet indices, and erythrocyte metrics, yielded values within normal physiological ranges, comparable to those recorded in the untreated control group. Taken together, these findings support the overall tolerability of the extracts and reinforce their potential for further therapeutic development. A comparative analysis of our molecular docking results with those reported in three referenced studies highlights both similarities and differences in how natural compounds interact with HMG-CoA reductase, the enzyme central to cholesterol biosynthesis. In our investigation, hesperidin and rutin, bioactive constituents from *Zingiber officinale* and *Citrus limon*, demonstrated strong binding affinities of −10.4 kcal/mol and −9.0 kcal/mol, respectively, reflecting a high potential for enzymatic inhibition. These results are consistent with the findings reported by Medina-Franco and colleagues [99], who reported that α-asarone binds HMG-CoA reductase with a binding energy of −6.60 kcal/mol, primarily via hydrogen bonds involving Lys-691 and Glu-559. In a similar vein, Lin et al. identified curcumin and salvianolic acid C as potent inhibitors of the same enzyme, as evidenced by favorable docking scores and experimentally determined IC₅₀ values of 4.3 µM and 8 µM, respectively [100].

Our molecular modeling results suggest that hesperidin and 6-gingerol are situated within the catalytic site of HMG-CoA reductase, engaging with key amino acid residues such as Lys-735 and Glu-559. These residues play a fundamental role in substrate binding and catalytic activity. The presence of both hydrogen bonding and hydrophobic interactions supports the notion that these compounds function through a competitive inhibition mechanism, comparable to that of established statins such as atorvastatin [101,102]. This interaction profile holds particular pharmacodynamic relevance, as it suggests a consistent and predictable inhibitory effect, thereby enhancing the translational value of our findings. In contrast to allosteric modulators, which may result in variable efficacy or partial inhibition, competitive inhibitors such as hesperidin and 6-gingerol are more likely to produce reliable and reproducible therapeutic outcomes [102,103].

Further perspective is offered by the study conducted by Jensi et al., which demonstrated that quercetin and leucocyanidin can form stable hydrogen bonds with HMG-CoA reductase, resulting in notable reductions in LDL cholesterol in animal models. While 6-gingerol in our investigation showed a moderate binding energy of −6.1 kcal/mol, the higher affinities observed for hesperidin and rutin highlight their superior inhibitory potential relative to quercetin and leucocyanidin. These findings strengthen the case for considering hesperidin and rutin as promising lead compounds for future drug development and optimization efforts [104].

The work of Jensi et al. provides additional insight, showing that quercetin and leucocyanidin can form stable hydrogen bonds with HMG-CoA reductase and producing meaningful reductions in LDL cholesterol levels in animal models. In comparison, although 6-gingerol in our study exhibited a moderate binding energy of −6.1 kcal/mol, the markedly stronger affinities demonstrated by hesperidin and rutin underscore their enhanced inhibitory potential. These results reinforce the value of hesperidin and rutin as promising molecular candidates for future drug refinement and development strategies targeting cholesterol regulation [105].

A key strength of this study is its emphasis on the synergistic effects arising from the combination of ginger and lemon extracts, a dimension overlooked mainly in the studies cited for comparison. This combined approach not only yielded stronger binding affinities but also led to more pronounced improvements in lipid parameters, including significant reductions in LDL cholesterol and elevations in HDL cholesterol, compared to treatments using individual compounds. These findings highlight the therapeutic value of synergy in phytomedicine and emphasize the potential of multi-component formulations to enhance clinical efficacy.

The flavonoids and gingerols contained in the *Zingiber officinale* and *Citrus limon* juice blend appear to work synergistically to reduce lipid levels by targeting critical metabolic pathways, while also exhibiting a favorable safety profile, as confirmed by our toxicity assessments. Nevertheless, it is important to acknowledge that the Triton WR-1339-induced hyperlipidemia model, although well-established and commonly employed in preclinical studies, induces lipid abnormalities acutely by inhibiting lipoprotein lipase. This mechanism contrasts with the chronic, diet-related dyslipidemia more typically observed in human pathophysiology [28,106]. This distinction warrants caution when extrapolating the current findings to clinical settings, and future studies would benefit from incorporating diet-induced or genetically modified models that more closely mimic the human pathophysiology of hyperlipidemia. In addition, the limited systemic bioavailability of key phytochemicals, such as gingerols and shogaols, remains a significant challenge for clinical applications. These compounds are hindered by poor solubility in aqueous environments, rapid first-pass metabolism, and low intestinal uptake, all of which contribute to a short plasma half-life and subtherapeutic concentrations *in vivo*. [107,108].

To overcome these challenges, several innovative drug delivery systems, such as nanoemulsions, liposomes, polymeric nanoparticles, and self-microemulsifying drug delivery systems (SMEDDS), have been explored to improve the solubility, physicochemical stability, and systemic bioavailability of plant-derived compounds [8,109]. In response to these limitations, various advanced drug delivery platforms, including nanoemulsions, liposomal formulations, polymer-based nanoparticles, and self-microemulsifying drug delivery systems (SMEDDS), have been investigated for their potential to enhance the solubility, structural stability, and bioavailability of bioactive plant compounds [8,80,109]. These innovative approaches embody a modern, systems-level vision of phytotherapeutic development and offer promising avenues for both translational and clinical research.

## 5. Conclusions

This study investigated the lipid-lowering effects of *Zingiber officinale* and *Citrus limon* juices, administered both individually and in combination, in a murine model of triton-induced hyperlipidemia. To support the experimental findings, an *in silico* analysis was also conducted to assess the anti-hyperlipidemic potential of key phytochemicals. Our results revealed that each juice, when administered separately, significantly reduced lipid levels. More notably, the combined treatment produced a more pronounced effect, suggesting a synergistic interaction between the bioactive constituents of *Zingiber officinale* and *Citrus limon*. Complementary molecular docking results indicated that several compounds derived from *Citrus limon*, including eriodictyol, rutin, hesperidin, and isorhamnetin, exhibit strong predicted affinity for HMG-CoA reductase, a central enzyme in cholesterol biosynthesis.

Collectively, these findings highlight the potential of *Zingiber officinale* and *Citrus limon* juices, used alone or in synergy, as promising natural agents for managing hyperlipidemia. Looking ahead, future research should explore the clinical relevance of these extracts in chronic dyslipidemia management, with particular attention to elucidating their underlying mechanisms of action. Moreover, advancing formulation strategies to optimize bioavailability will be essential for translating these findings into practical phytotherapeutic applications. Together, these insights contribute to a broader understanding of integrative approaches for controlling acute hyperlipidemia and offer a foundation for future investigations in the field of cardiovascular health.

## Supporting information

S1 File Excel file containing the raw data of all biological tests performed in this study. This file includes individual sheets for each assay: total phenolic content, total flavonoid content, antioxidant assays (DPPH, ABTS, TAC, and FRAP), MDA quantification, lipid profile (TC, HDL, LDL, TG), atherogenic index of plasma (AIP), calculated ratios (LDL/HDL, TC/HDL, TG/HDL), and toxicity studies (acute toxicity and subacute toxicity). Each test is presented in a separate and clearly labeled worksheet to ensure clarity and reproducibility.
(XLSX)

## Abbreviations

ABTS: 2,2-azino-bis (3-ethylbenzothiazoline-6-sulfonic acid)]
AIP: Atherogenic index of plasma
ATG: Atorvastatin-treated group
BHA: Butylated hydroxy anisole
CHOL: : Cholesterol
DPPH: 1,1-Diphenyl picryl hydrazil
DW: Dry weight
FTG: Formulation-treated group
GAE: Gallic acid equivelent
GJ: Ginger juice
GJTG: Ginger juice-treated group
HCG: Hypercholesterolemic group
HCT: Hematocrit
HDL: High-density lipoprotein
HFD: High-fat diet
HGB: Hemoglobin concentration
HPLC: High-performance liquid chromatography
IC50: Inhibitory concentration 50
LDL: Low-density lipoprotein
LJ: Lemon juice
LJTG: Lemon juice-treated group
MCH: Mean corpuscular hemoglobin
MCHC: Mean corpuscular hemoglobin concentration
MCV: Mean corpuscular volume
MDA: Malondialdehydes
MPV: Mean platelet volume
NCG: Normocholesterolemic group
PLT: Platelets
QE: Quercetin equivalent
RBC: Red blood cells
TAC: Total antioxidant capacity
TBARS: Thiobarbituric acid species
TC: Total cholesterol
TG: Triglycerides
VLDL: Very low-density lipoprotein
WBC: White blood cells.
